# TRIM32 Senses and Restricts Influenza A Virus by Ubiquitination of PB1 Polymerase

**DOI:** 10.1371/journal.ppat.1004960

**Published:** 2015-06-09

**Authors:** Bishi Fu, Lingyan Wang, Hao Ding, Jens C. Schwamborn, Shitao Li, Martin E. Dorf

**Affiliations:** 1 Department of Microbiology & Immunobiology, Harvard Medical School, Boston, Massachusetts, United States of America; 2 Department of Biochemistry and Medical Genetics, University of Manitoba, Winnipeg, Canada; 3 Luxembourg Centre for Systems Biomedicine, University of Luxembourg, Luxembourg; 4 Department of Physiological Sciences, Oklahoma State University, Stillwater, Oklahoma, United States of America; Mount Sinai School of Medicine, UNITED STATES

## Abstract

Polymerase basic protein 1 (PB1) is the catalytic core of the influenza A virus (IAV) RNA polymerase complex essential for viral transcription and replication. Understanding the intrinsic mechanisms which block PB1 function could stimulate development of new anti-influenza therapeutics. Affinity purification coupled with mass spectrometry (AP-MS) was used to identify host factors interacting with PB1. Among PB1 interactors, the E3 ubiquitin ligase TRIM32 interacts with PB1 proteins derived from multiple IAV strains. TRIM32 senses IAV infection by interacting with PB1 and translocates with PB1 to the nucleus following influenza infection. Ectopic TRIM32 expression attenuates IAV infection. Conversely, RNAi depletion and knockout of TRIM32 increase susceptibility of tracheal and lung epithelial cells to IAV infection. Reconstitution of *trim32^-/- ^* mouse embryonic fibroblasts with TRIM32, but not a catalytically inactive mutant, restores viral restriction. Furthermore, TRIM32 directly ubiquitinates PB1, leading to PB1 protein degradation and subsequent reduction of polymerase activity. Thus, TRIM32 is an intrinsic IAV restriction factor which senses and targets the PB1 polymerase for ubiquitination and protein degradation. TRIM32 represents a model of intrinsic immunity, in which a host protein directly senses and counters viral infection in a species specific fashion by directly limiting viral replication.

## Author Summary

Influenza A virus presents a continued threat to global health with considerable economic and social impact. Vaccinations against influenza are not always effective, and many influenza strains have developed resistance to current antiviral drugs. Thus, it is imperative to find new strategies for the prevention and treatment of influenza. Influenza RNA-dependent RNA polymerase is a multifunctional protein essential for both transcription and replication of the viral genome. However, we have little understanding of the mechanisms regulating viral RNA polymerase activity or the innate cellular defenses against this critical viral enzyme. We describe how the E3 ubiquitin ligase, TRIM32, inhibits the activity of the influenza RNA polymerase and defends respiratory epithelial cells against infection with influenza A viruses. TRIM32 directly senses the PB1 subunit of the influenza virus RNA polymerase complex and targets it for ubiquitination and proteasomal degradation, thereby reducing viral polymerase activity.

## Introduction

Influenza A virus A (IAV) is a human respiratory pathogen that causes seasonal epidemics and occasional global pandemics with devastating levels of morbidity and mortality. IAV is a member of the *Orthomyxoviridae* family and possesses eight segments of negative-sense single-stranded RNA genome. Replication and transcription of these IAV segments is catalyzed by a heterotrimeric RNA-dependent RNA polymerase complex, which consists of an acidic subunit (PA) and two basic subunits, PB1 and PB2 [1,2].

PB1 is the structural backbone for formation of the IAV polymerase complex [1]. PB1 contains a 14 residue binding site for PA at the N-terminus and a C-terminal domain for PB2 association [3–6]. Since the activity of RNA-dependent polymerases is distinct from enzymes found in host cells, these viral proteins are promising drug targets for interfering with viral replication [7,8]. Little is understood about the natural defenses employed by host cells to defend against the IAV polymerase. In this report, we analyze PB1 protein complexes and find a host interactor, tripartite motif-containing protein 32 (TRIM32), which directly targets PB1 proteins to restrict influenza virus replication.

TRIM32 was initially identified as a protein that binds HIV-1 tat (a key transactivator of viral transcription) [9,10]. TRIM32 contains an N-terminal signature tripartite motif (TRIM) consisting of RING, B-box and coiled-coil domains followed by a spacer segment and a series of NHL repeats. The presence of the RING domain is a sign that TRIM family proteins may function as ubiquitin E3 ligases, catalyzing transfer of ubiquitin from an E2 enzyme to form a covalent bond with a substrate lysine. Genetic mutation in the TRIM32 NHL domains causes recessive hereditary muscle disorders, often with a neurogenic component, including limb girdle muscular dystrophy 2H and sarcotubular myopathy [11–14]. These conditions are phenocopied in knockout mice that lack *trim32* [15,16] and knockin animals that carry a disease associated TRIM32 mutation [17]. In addition, mutations in the TRIM32 B-box domain are responsible for Bardet Biedl syndrome, which has a pleiotropic phenotype often accompanied with retinal degeneration [18,19].

TRIM32 is a ubiquitously expressed E3 ligase, which targets several proteins for ubiquitination, including actin [20], PIASγ [21], Abl-interactor 2 [22], c-Myc [23], PKCζ [24], dysbindin [25], X-linked inhibitor of apoptosis (XIAP) [26], desmin filaments [27], p73 transcription factor [28], STING [29] and Gli-related Krüppel-like zinc finger protein (Glis2) [30]. Based on this broad substrate specificity, it is not surprising that TRIM32 has versatile activities and is linked to diverse biological processes, including innate immunity [31,32], development and differentiation [15,16,23,33,34], regulation of microRNA [23,35], and tumorigenesis [36,37]. However, the role of TRIM32 in intrinsic immunity and viral restriction remains enigmatic. This report characterizes a role for TRIM32 in intrinsic cellular defense against influenza viruses by targeting the influenza polymerase for ubiquitination and degradation.

## Results

### TRIM32 interacts and translocates with PB1 following IAV infection

Mass spectrometry was used to examine the physical interactions between IAV PB1 and endogenous cellular proteins. PB1 protein complexes were immunoaffinity purified from HEK293 cells stably expressing FLAG tagged PB1 derived from the influenza A/Puerto Rico/8/1934 (PR8). Two independent purifications were analyzed by LC/MS-MS analysis. Controls include our laboratory database of 200 FLAG-tagged non-viral proteins isolated by identical procedures from stably transfected HEK293 cell lines [32,38,39]. A well-established computational algorithm, known as SAINT, was applied to the dataset [40,41]. Twenty-six proteins had SAINT scores above 0.89 and were designated high confidence interacting proteins (HCIP), including 18 proteins (S1 Table) reported to associate with PB1 or IAV polymerase complexes [42–44].

**Fig 1:**
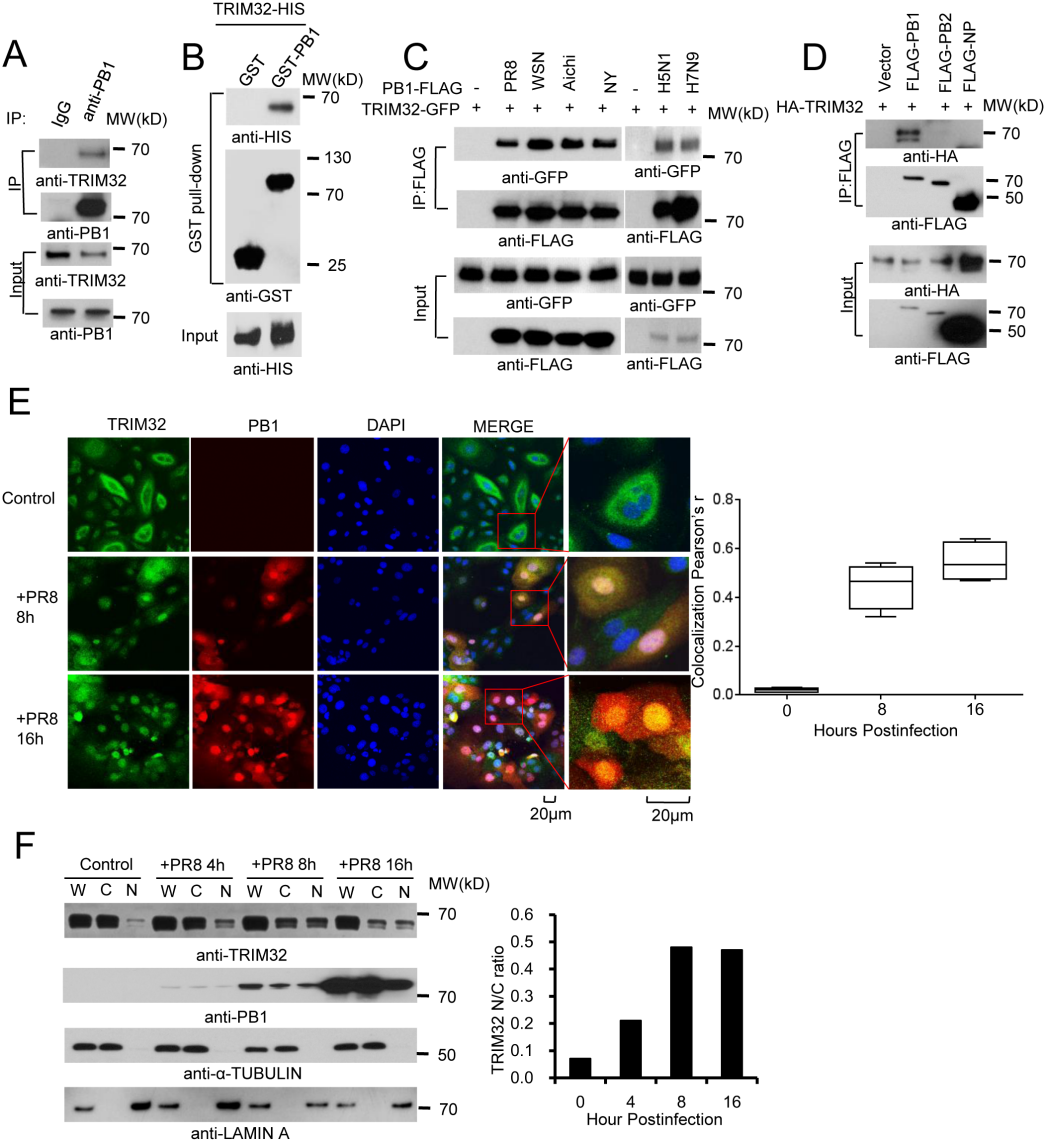
TRIM32 interacts and translocates with influenza A virus PB1 protein. (A) Primary human tracheal epithelial cells were infected with 0.1 MOI PR8 IAV for 16 hr. Whole cell lysates (WCL) were subjected to immunoprecipitation (IP) and immunoblotting with indicated antibodies to detect endogenous interactions. Molecular weights (MW) are indicated. (B) GST pull down of bacterially expressed GST-PB1 and HIS-TRIM32. (C) FLAG-tagged PB1 from 6 different influenza A strains [PR8, A/Puerto Rico/8/1934 (H1N1); WSN, A/WSN/1933 (H1N1); Aichi, A/Aichi/2/1968 H3N2; NY, A/New York/1682/2009 (H1N1); A/Vietnam/1194/2004 (H5N1); A/Anhui/1/2013 (H7N9)] were co-expressed with TRIM32-GFP in HEK293 cells. After 48 hr, cell lysates were immunoprecipitated with anti-FLAG and probed as indicated. As input controls, WCL were immunoblotted. (D) TRIM32 fused with HA epitope was co-transfected into HEK293 cells with PR8 derived FLAG-tagged PB1, PB2 or NP. After 48 hr, WCL were immunoprecipitated with anti-FLAG antibody and blotted with indicated reagents. TRIM32 can appear as a doublet on Western blots. (E) Primary human tracheal epithelial cells were infected with 0.01 MOI PR8 strain IAV for 8 or 16 hr and stained with anti-TRIM32 (green), anti-PB1 (red) and DAPI nuclear stain (blue). Right panel shows quantitated TRIM32-PB1 colocalization data. (F) A549 cells were infected with 0.01 MOI IAV PR8 strain for the indicated times, whole cell lysates (W) or cytosolic (C) and nuclear (N) fractions were extracted and blotted as indicated. Right panel depicts the densitometric ratio of nuclear to cytoplasmic PB1.

In a preliminary screen, 6 available GFP- or FLAG-tagged HCIP (TRIM32, STUB1, IQSEC, GALK, PDCD6 and HAUS6/DGT6) were expressed in HEK293 cells and examined for their effects on viral replication using the PR8-Gaussia luciferase reporter virus [45]. The most potent inhibition of IAV replication was noted following ectopic expression of TRIM32 (S1A Fig). Interactions between TRIM32 and IAV proteins had not been previously reported. Thus, we first validated the protein interaction. Following PR8 IAV infection of either primary human tracheal epithelial cells or HEK293 cells, endogenous TRIM32 binds to viral PB1 (Figs 1A and S1B). To demonstrate this interaction is direct, TRIM32-HIS and PB1-GST were purified from bacteria. *In vitro* GST pull down assays confirmed the direct association of PB1 with TRIM32 (Fig 1B). We next examined if TRIM32 can associate with PB1 proteins derived from different IAV strains. PB1 proteins from 6 IAV strains [PR8, A/Puerto Rico/8/1934 (H1N1); WSN, A/WSN/1933 (H1N1); NY, A/New York/1682/2009 (H1N1); Aichi, A/Aichi/2/1968 (H3N2); A/Vietnam/1194/2004 (H5N1) and A/Anhui/1/2013 (H7N9)] were co-transfected with TRIM32-GFP into HEK293 cells. Co-precipitation was noted between TRIM32 and PB1 from all 6 IAV strains (Fig 1C). Furthermore, the IAV ribonucleoprotein components PA, PB2 and NP failed to interact with TRIM32, demonstrating the specificity of PB1-TRIM32 interaction (Figs 1D and S1C). Taken together, TRIM32 physically interacts with PB1 polymerases derived from multiple IAV strains.

As influenza viruses are intrinsically sensitive to the antiviral action of interferons (IFN), we speculated that IFN might regulate TRIM32 expression. To address this issue, we infected A549 lung epithelial cells with PR8 IAV or treated the cells with IFN. TRIM32 mRNA and protein expression were then examined. However, there is no evidence that IAV infection or IFN stimulation modulate TRIM32 RNA or protein levels (S1D and S1E Fig). We conclude TRIM32 is constitutively expressed in A549 human lung epithelial cells, a model host cell line for IAV infection.

To examine TRIM32-PB1 co-localization, we infected primary human tracheal and A549 lung epithelial cells with PR8 IAV. In the absence of viral infection, endogenous TRIM32 is diffusely expressed in cytosolic foci and lesser amounts are detected in the nucleus. Following IAV infection TRIM32 accumulates in the nucleus (Figs 1E and S2A). Similarly, nuclear accumulation of endogenous TRIM32 is noted in A549 cells stably transfected with PB1 (S2B Fig). Influenza A driven TRIM32 nuclear translocation was biochemically confirmed by isolation of nuclear and cytosolic fractions (Fig 1F), indicating that IAV infection triggers translocation of TRIM32 to the nucleus within 4 hr. The evolutionarily conserved CRM1 (exportin 1) receptor is responsible for nuclear export of most proteins [46]. To examine the role of CRM1 in TRIM32 nuclear distribution, A549 cells were treated with CRM1 inhibitor leptomycin B. Addition of leptomycin B causes accumulation of nuclear TRIM32 in the absence of viral infection (S2C Fig), thereby implying that TRIM32 physiologically shuttles between the cytosol and nucleus. The combined data suggest that during IAV replication PB1 retains TRIM32 in the nuclear compartment.

### Domains required for TRIM32-PB1 interaction

**Fig 2:**
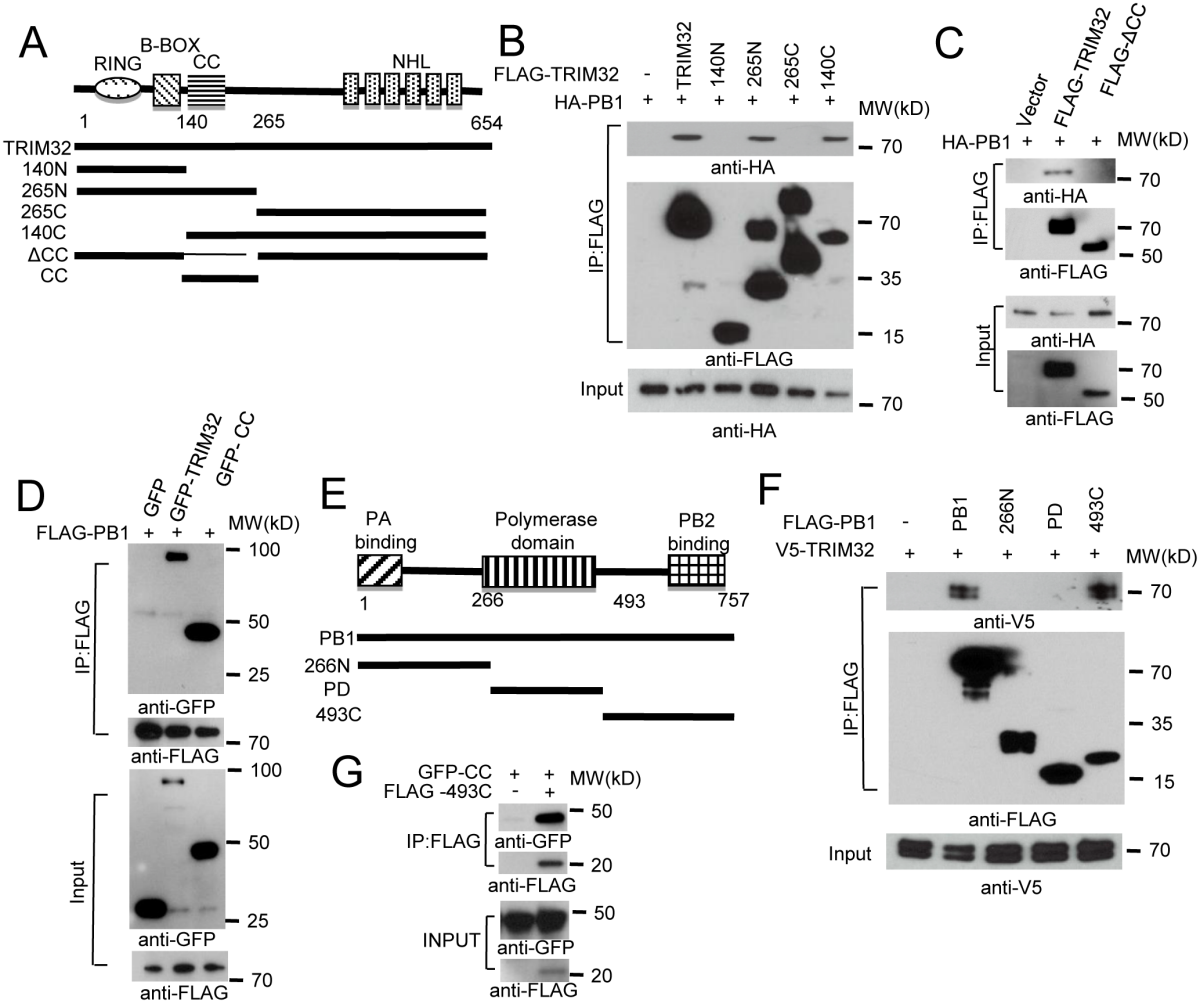
Domain requirements for TRIM32-PB1 interaction. (A) Schematic representation of TRIM32 protein domains and the individual TRIM32 deletion mutants investigated in this study. (B) Full length and various TRIM32 deletion mutants were fused with FLAG epitope and co-transfected with HA-PB1 into HEK293 cells. WCL were immunoprecipitated with anti-FLAG antibody and blotted with indicated reagents. (C) Full length and TRIM32 with a deleted CC segment were fused with FLAG epitope and co-transfected with HA-PB1 into HEK293 cells. WCL were immunoprecipitated with anti-FLAG antibody and blotted with indicated reagents. (D) Full length and TRIM32 CC-containing segment (residues 140–265) were tagged with GFP and co-transfected with FLAG-PB1 into HEK293 cells. WCL were immunoprecipitated with anti-FLAG antibody and blotted with the indicated reagents. (E) Schematic representation of IAV PB1 protein domains and PB1 deletion mutants investigated in this study. (F) Full length and various PB1 mutants (PR8) were fused with FLAG epitope and co-transfected with V5-TRIM32 into HEK293 cells. WCL were immunoprecipitated with anti-FLAG antibody and blotted with indicated reagents. (G) TRIM32 CC fragment (residues 140–265) was cotransfected with C-terminal PB1 fragment (residues 493–757) into HEK293 cells. Immunoprecipitation and immunoblotting were performed with indicated reagents.

To determine which segment of TRIM32 mediates its interaction with PB1, we generated a series of TRIM32 deletion mutants (Fig 2A). Initial experiments suggested TRIM32 constructs carrying residues 140 to 265 were involved in PB1 binding (Fig 2B). The 140 to 265 segment contains the TRIM32 CC domain plus part of the linker region. Deletion of this segment results in the loss of PB1 binding (Fig 2C), while this segment alone is sufficient for PB1 association (Fig 2D). Thus, TRIM32 residues 140 to 265 are necessary and sufficient for PB1 binding.

Assembly of the heterotrimeric IAV polymerase requires interactions between the PB1 N-terminal domain with the PA chain while the PB1 C-terminus associates with the polymerase PB2 protein [6,47,48]. PB1 truncation mutants were prepared in order to define the PB1 segment which interacts with TRIM32 (Fig 2E). The PB1 C-terminal region consisting of residues 493 to 757 was sufficient for interaction with TRIM32 (Fig 2F) and the TRIM32 CC-linker fragment (Fig 2G). These findings led us to question whether TRIM32 could compete with PB2 for PB1 binding. The failure of TRIM32 to block PB1-PB2 association suggests TRIM32 is not a competitive inhibitor of IAV polymerase assembly (S2D Fig). Fine mapping of IAV polymerase interaction sites suggest a stretch of 15 amino acids in the PB1 C-terminus are most important for PB2 binding, leaving an expansive “thumb” surface available for interactions with other proteins [1,2]. The combined data identify the essential peptide fragments for TRIM32-PB1 interaction.

### TRIM32 defends against influenza virus infection

**Fig 3:**
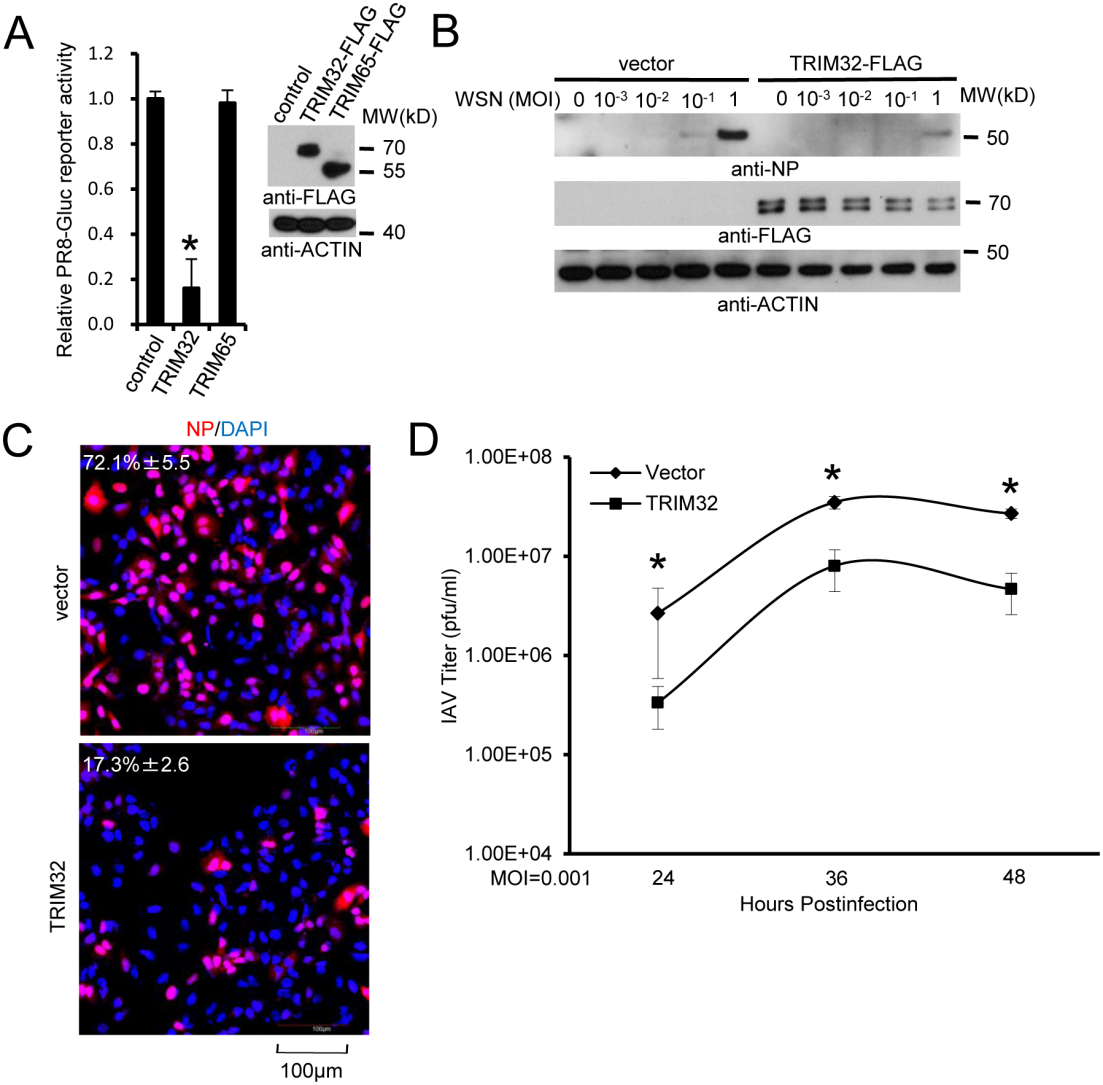
TRIM32 attenuates influenza A virus infection. (A) A549 cells were transfected with control vector, TRIM32-FLAG or TRIM65-FLAG. After 36 hr cells were infected with 0.01 MOI PR8-Gluc for 16 hr and then Gaussia luciferase activity was examined. An asterisk indicates P<0.01. (B) A549 stable cell lines carrying vector or TRIM32-FLAG were infected with indicated MOI of WSN strain IAV for 16 hr. WCL were blotted with indicated reagents. (C) A549 cells stably transfected with control vector or TRIM32-FLAG were infected with 0.01 MOI PR8 for 8 hr and stained with anti-NP (red) and DAPI (blue). The percentage of NP stained cells is indicated. (D) A549 stable cell lines transfected with vector or TRIM32-FLAG were infected with 0.001 MOI of WSN IAV for the indicated times. Supernatant was titered on MDCK cells and plaques were enumerated. Asterisk indicates P<0.05.

Four assay systems (reporter assay, Western blot, immunofluorescence and plaque assay) were used to evaluate the biological effect of TRIM32 overexpression on IAV replication. First, FLAG-tagged TRIM32 and TRIM65 (a control TRIM family member with established E3 ligase activity [39,49]) were transfected into A549 cells followed by infection with PR8-Gaussia luciferase reporter virus. TRIM32 selectively restricted IAV replication (Fig 3A) without significantly impacting cell viability (S3A Fig). We then determined the effect of TRIM32 on viral infection by examining IAV NP protein expression using Western blot and immunofluorescence. A549 lung epithelial cells stably transfected with TRIM32 were infected with WSN and PR8 IAV. TRIM32 overexpression resulted in viral restriction as noted by decreased levels of NP protein detected by Western blot (Figs 3B and S3B). Similarly, HEK293 cells transfected with FLAG-TRIM32 or GFP-TRIM32 displayed decreased levels of viral protein production (S3C and S3D Fig). Microscopic inspection confirmed that TRIM32 overexpression inhibited PR8 and WSN viral NP protein expression (Figs 3C and S3E). Plaque assays were used to determine the effects of TRIM32 on production of infectious IAV particles. Stable or transient overexpression of TRIM32 consistently reduced viral titers (Figs 3D and S3F). To compare the relative susceptibility of various IAV strains to TRIM32-mediated restriction, A549 stable cell lines transfected with control vector or TRIM32-FLAG were infected with 0.001 MOI of 4 different IAV strains. After 18 hr, supernatants were transferred to fresh A549 target cells and the relative fraction of infected cells was determined. TRIM32 transfection results in 70–80% reduction of infectious IAV particles (S3G Fig). Thus, the combined findings indicate TRIM32 is an IAV restriction factor.

### TRIM32 deficiency increases susceptibility to influenza infection

**Fig 4:**
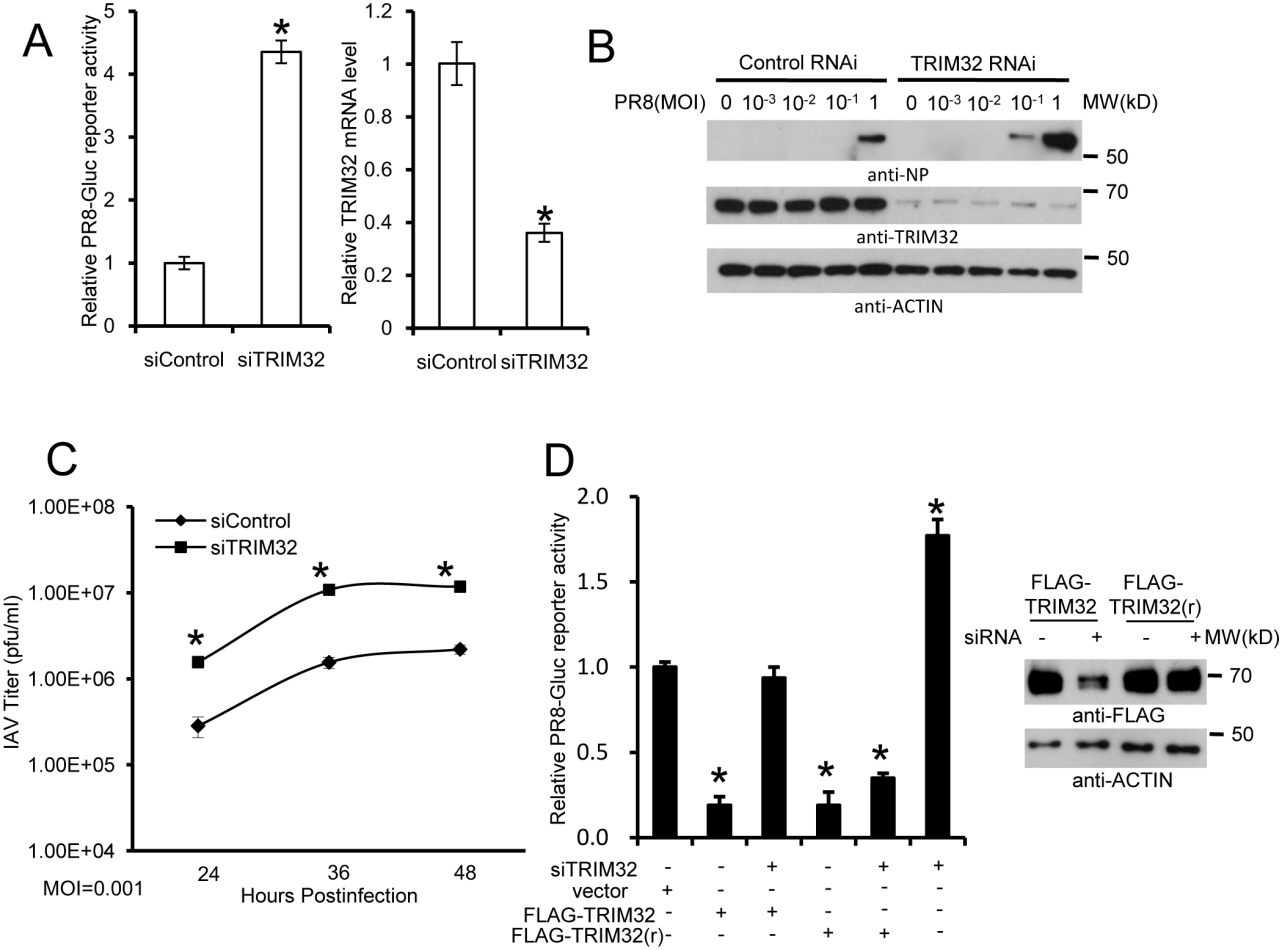
RNAi depletion of TRIM32 promotes influenza virus infection. (A) Primary human tracheal epithelial cells were transfected with scrambled control or TRIM32 siRNA duplex #1. After 24 hr cells were infected with 0.01 MOI PR8-Gluc for 16 hr. The relative luciferase activity was examined. An asterisk indicates P<0.01. Right panel displays knockdown efficiency by qPCR. (B) A549 cells were transfected with control siRNA or TRIM32 siRNA#1. After 24 hr cells were infected with indicated MOI of PR8 IAV. WCL were blotted with the indicated antibodies. (C) Tracheal epithelial cells transfected with control or TRIM32 siRNA were infected with 0.001 MOI of WSN IAV for the indicated times. Culture supernatants containing IAV were titered on MDCK cells and plaques were enumerated. An asterisk indicates P<0.05. (D) HEK293 cells were transfected with TRIM32 siRNA and wild type TRIM32 or a TRIM32 rescue construct. After 24 hr cells were infected with 0.01 MOI PR8-Gluc for 16 hr. The relative luciferase signal is shown. An asterisk indicates P<0.05. Western blot shows knockdown efficiency.

To complement the above overexpression data, we depleted TRIM32 with siRNA. Decreased TRIM32 expression correlated with increased IAV reporter activity in primary respiratory epithelial cells (Fig 4A) and also in HEK293 cells (S4A Fig). In addition, silencing TRIM32 enhanced IAV propagation in A549 cells, as detected by plaque assay and increased NP levels (Figs 4B, S4B and S4C). Knockdown of TRIM32 also enhanced IAV propagation in primary tracheal epithelial cells, as detected by plaque assay (Fig 4C). To exclude off-target effects of siRNA, cells were transfected with wild type TRIM32 or a siRNA resistant TRIM32 rescue construct before infection with PR8 reporter virus. Cells transfected with wild type or rescue TRIM32 constructs displayed comparable levels of TRIM32 expression and both decreased viral replication as demonstrated by reduced reporter activity (Fig 4D). Importantly, when combined with TRIM32 siRNA the rescue construct restored antiviral activity, validating siRNA specificity (Fig 4D).

**Fig 5:**
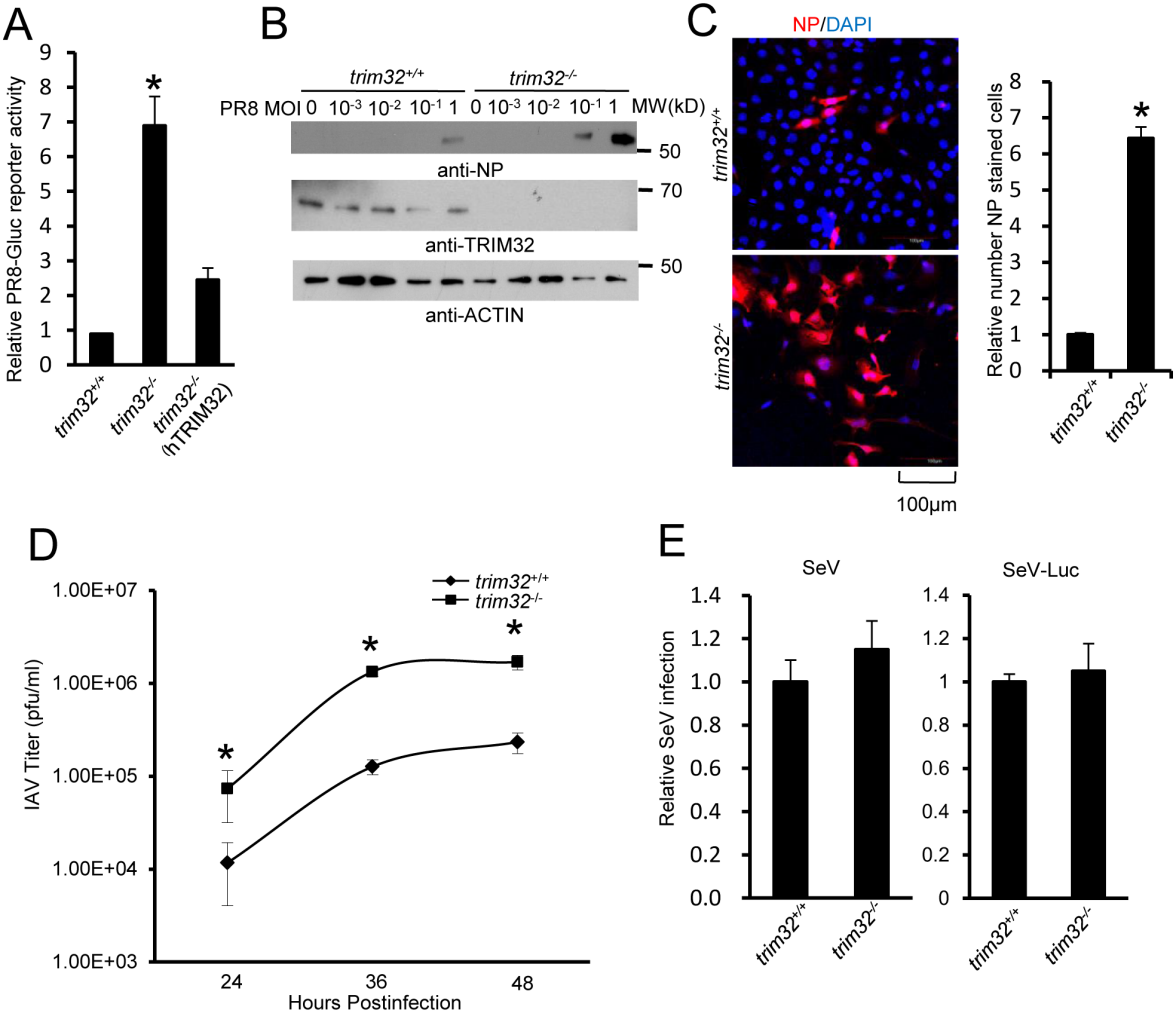
TRIM32 deficiency increases susceptibility to influenza A virus infection. (A) *Trim32* ^*+/+*^, *trim32* ^-/-^ and *trim32* ^*-/-*^ MEF transfected (24 hr) with human TRIM32 were infected with 0.1 MOI PR8-Gluc. The relative luciferase signal is shown. An asterisk indicates significant difference (P<0.05) for *trim32* ^*-/-*^ vs. either *trim32* ^+/+^ or TRIM32 reconstituted *trim32* ^-/-^ MEF. (B) *Trim32* ^+/+^ and *trim32* ^-/-^ MEF were infected with indicated MOI of PR8 IAV. After 16 hr, WCL were blotted with indicated reagents. (C) *Trim32* ^+/+^ and *trim32* ^-/-^ MEF cells were infected with PR8 IAV for 8 hr, then cells were stained with anti-NP (red) and DAPI (blue). The right panel shows the relative ratio of NP stained cells. An asterisk indicates P<0.01. (D) *Trim32* ^+/+^ and *trim32* ^-/-^ MEF were infected with 0.01 MOI of WSN IAV for the indicated times. Supernatants were titered on MDCK cells and pfu were enumerated. An asterisk indicates P<0.05. (E) *Trim32* ^+/+^ and *trim32* ^-/-^ MEF were infected with wild type Sendai virus (SeV) or luciferase reporter Sendai virus (SeV-Luc) for 16 hr. Sendai infection was detected by staining with anti-Sendai antibody or luciferase assay. Relative numbers of Sendai virus infected cells or luciferase activities are presented.

We next examined IAV infection in TRIM32 deficient mouse embryonic fibroblasts (MEF). A 7 fold increase in PR8 luciferase reporter activity was noted in *trim32* ^-/-^ MEF compared to wild type controls and antiviral activity was partially rescued by transfection with human TRIM32 (Fig 5A). In line with these results approximately 10-fold enhancement of viral NP protein expression was observed after PR8 infection of *trim32* ^-/-^ MEF (Fig 5B) and significantly more *trim32* ^*-/-*^ cells were stained with anti-NP antibody (Fig 5C). We also evaluated the impact of TRIM32 on viral propagation using plaque assays. As predicted, *trim32* deficient cells produce more infectious viral particles than control MEF (Fig 5D). Finally, TRIM32 deficient and control MEF were infected with 0.1 MOI of 4 different IAV strains. Supernatants were transferred to A549 target cells and the relative fraction of infected cells was determined. *Trim32* ^-/-^ cells consistently produced 3 to 6 fold more infectious virus than control cells (S4D Fig). To establish virus restriction specificity, MEF were infected with Sendai virus. TRIM32 deficiency did not alter the level of infection with wild type Sendai or a Sendai-luciferase reporter virus (Fig 5E). The combined data using *trim32* deficient cells indicate TRIM32 is an antiviral cellular factor that acts to curb infections with influenza viruses.

### E3 ligase activity of TRIM32 is required for IAV restriction

TRIM32 was reported to regulate NF-κB and IFN driven cytokine production, which may mediate the antiviral activity of TRIM32 [29,32,50–52]. Thus, we investigated the capacity of *trim32* ^+/+^ and *trim32* ^-/-^ MEF to produce IFNβ after infection with PR8 IAV (ΔNS1) virus [53] or treatment with the synthetic analog of dsRNA, poly(I:C). *Trim32* ^*+/+*^ and trim*32* ^-/-^ MEF show comparable levels of IFNβ mRNA after IAV infection or poly(I:C) stimulation (S5A and S5B Fig). Furthermore, similar levels of IAV reporter activity were noted in MEF derived from wild-type and NF-κB deficient *p65* ^*-/-*^ mice (S5C Fig). The results suggest that TRIM32 does not alter influenza-activated IFN production and indicate other mechanisms are needed to account for TRIM32-mediated IAV restriction.

**Fig 6:**
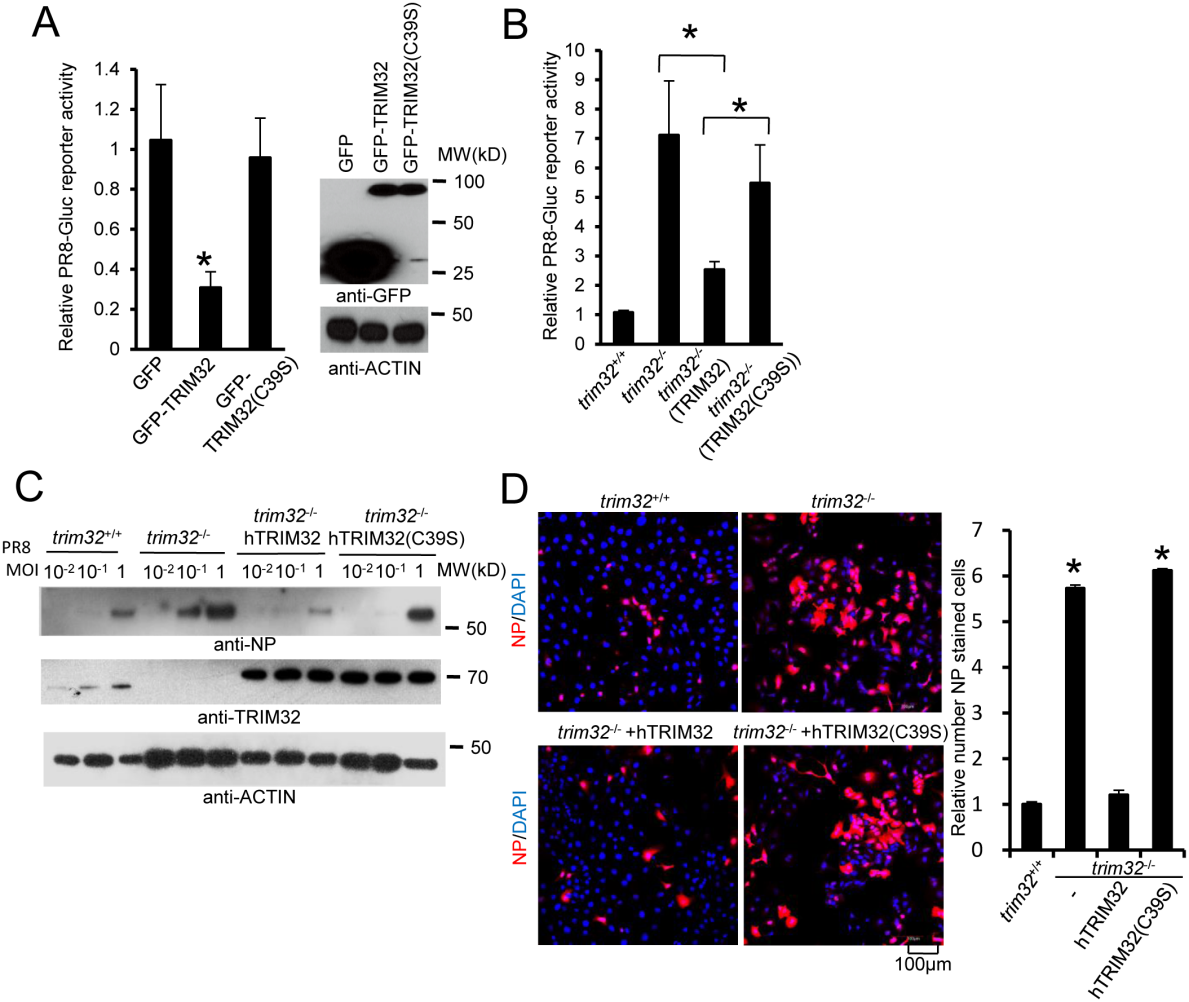
E3 ligase activity is indispensable for TRIM32-dependent against influenza A virus. (A) HEK293 cells were transfected with GFP, GFP-TRIM32 or GFP-TRIM32(C39S). After 24 hr, cells were infected with 0.01 MOI PR8-Gluc for reporter assay. The relative luciferase signal is shown. Asterisk indicates P<0.05 (GFP-TRIM32 vs. either GFP or GFP-TRIM32(C39S) transfected cells). (B) *Trim32* ^+/+^, *trim32* ^-/-^ or *trim32* ^-/-^ MEF reconstituted with TRIM32 and TRIM32(C39S) were infected with 0.1 MOI PR8-Gluc. The relative luciferase signal is shown. An asterisk indicates P<0.05. (C) *Trim32* ^+/+^, *trim32* ^-/-^ or *trim32* ^-/-^ MEF reconstituted with TRIM32 and TRIM32 (C39S) were infected with designated MOI of IAV PR8 for 16 hr. WCL were blotted with indicated reagents. (D) *Trim32* ^+/+^, *trim32* ^-/-^ or *trim32* ^-/-^ reconstituted with TRIM32 and TRIM32(C39S) MEF were infected with 0.1 MOI PR8 for 16 hr, then cells were stained with anti-NP (red) and DAPI. The right panel shows the relative ratio of NP stained cells. An asterisk indicates P<0.01.

RING domains are critical for E3 ligase function in TRIM family molecules. Thus, we evaluated the role of the TRIM32 RING domain in antiviral activity. The TRIM32(C39S) mutation disrupts the rigid cross-braced architecture of the RING domain and destroys E3 ligase activity [29]. Antiviral activity was measured after transfection of HEK293 cells with TRIM32 or the TRIM32(C39S) mutant. Wild type TRIM32 attenuates infection with the IAV reporter virus. In contrast, ectopic expression of the TRIM32(C39S) mutant protein failed to defend against IAV infection (Fig 6A). Reconstitution of *trim32* ^-/-^ MEF with human TRIM32 restores host restriction of PR8 reporter virus, while reconstitution with the TRIM32(C39S) mutant retains high viral propagation (Fig 6B). Similarly, the TRIM32(C39S) mutant failed to restore antiviral activity in *trim32* ^-/-^ MEF as assayed by Western blotting and immunofluorescence for viral NP proteins (Fig 6C and 6D). Thus, we conclude that E3 ligase activity is critical for the antiviral activity of TRIM32.

### TRIM32 ubiquitination of PB1 leads to its protein degradation

**Fig 7:**
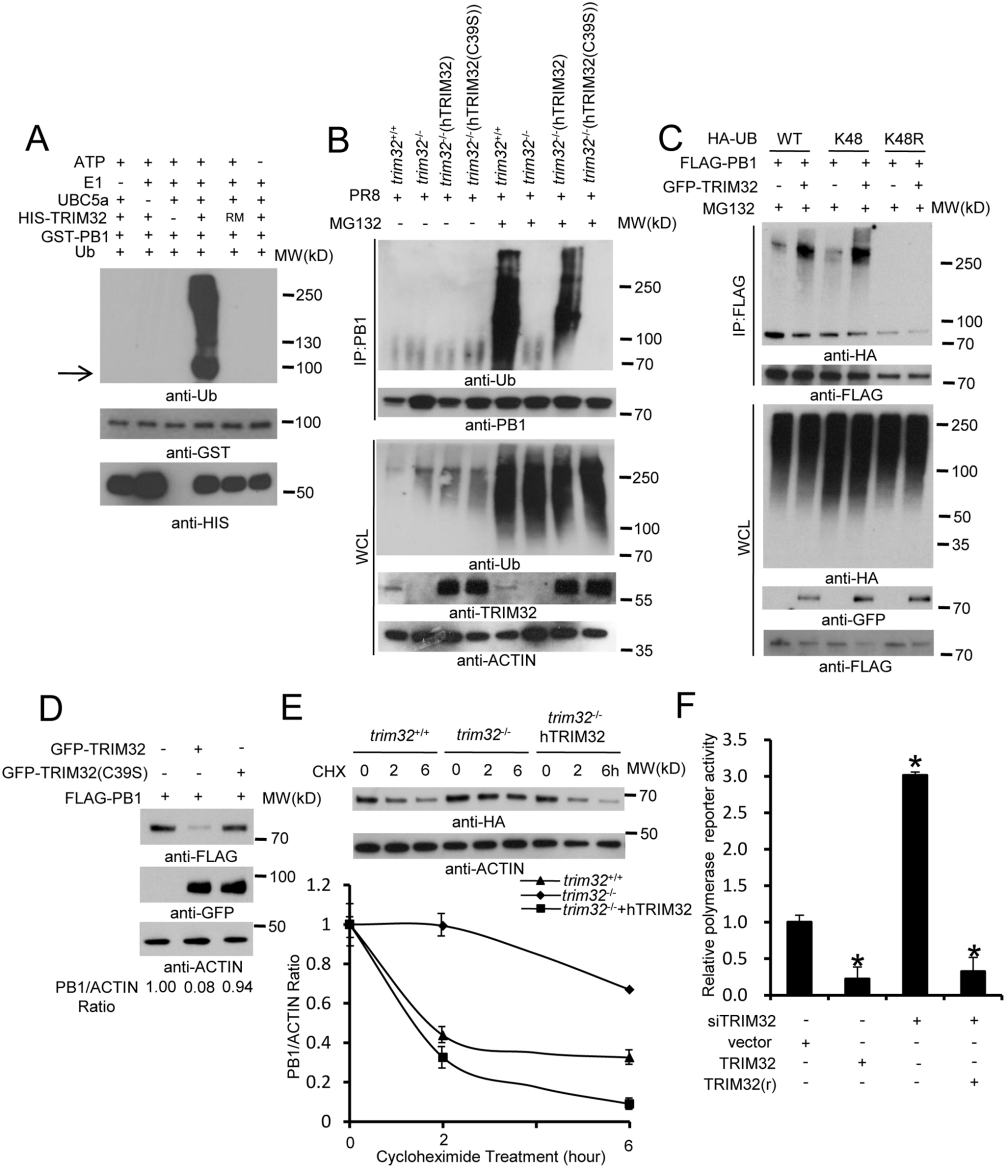
TRIM32 limits viral infection by targeting PB1 for ubiquitination. (A) *In vitro* ubiquitination of PB1 by TRIM32 plus E1, E2 (UBCH5A), ATP and ubiquitin (Ub). GST-tagged PB1, HIS-tagged TRIM32 and the catalytically dead TRIM32(C39S) RING mutant (RM) were purified from bacteria. The arrow indicates approximate PB1 position. (B) *Trim32* ^+/+^, *trim32* ^-/-^ or TRIM32 and TRIM32(C39S) reconstituted *trim32* ^-/-^ MEF were infected with 0.1 MOI PR8 IAV for 16 hr, then treated with 10 μg/ml MG132. After 6 hr cell lysates were immunoprecipitated with anti-PB1 and probed as indicated. Whole cell lysates are presented as input controls. (C) HEK293 stably transfected cell lines carrying FLAG-PB1 were transiently transfected with either HA-tagged wild type, K48 only or K48R ubiquitin along with GFP-TRIM32. Cells were treated for 6 hr with 10 μg/ml MG132, then immunoprecipitated with anti-FLAG and probed with the indicated antibodies. (D) GFP-TRIM32 or TRIM32(C39S) were transfected into HEK293 cells stably transfected with FLAG-PB1. Immunoblotting was performed with indicated reagents. Quantitative Western blotting was used to determine PB1 levels, which were used to calculate the ratio of PB1 to actin. (E) *Trim32* ^+/+^, *trim32* ^-/-^ and *trim32* ^-/-^ MEF reconstituted with TRIM32 were transfected with PB1-HA for 48 hr, then treated with 20 μg/ml cycloheximide for 0, 2 or 6 hr. WCL were blotted with indicated reagents. Quantitative Western blotting was used to derive data presented in lower panel. (F) HEK293 cells were transiently transfected with a plasmid cocktail containing PR8 PB1, PB2, PA, NP expression plasmids plus a polymerase I plasmid expressing an influenza virus-like RNA coding for the reporter protein firefly luciferase, Renilla luciferase control and TRIM32 siRNA along with TRIM32 wild type or rescue mutant for 48 hr. The relative luciferase signal is shown. An asterisk indicates P<0.05.

To determine whether TRIM32 can directly couple ubiquitin onto PB1, bacterially derived TRIM32 and PB1 were combined in an *in vitro* ubiquitination assay. TRIM32 is able to heavily conjugate ubiquitin onto PB1 *in vitro* (Fig 7A). PB1 ubiquitination is dependent on the presence of ubiquitin plus an E1, E2, and an ATP regenerating system. The TRIM32(C39S) RING mutant (RM) fails to conjugate ubiquitin onto PB1 (Fig 7A). To examine the role of TRIM32 on PB1 ubiquitination within cells, *trim32* ^-/-^ MEF stably transfected with either TRIM32 or TRIM32(C39S) were infected with PR8 IAV. After treatment with the proteasomal inhibitor, MG132, PB1 was immunoprecipitated. Ubiquitinated PB1 showing a characteristic high molecular weight ladder was noted from *trim32* ^*+/+*^ MEF or *trim32* ^-/-^ MEF reconstituted with TRIM32 (Fig 7B). Ubiquitinated PB1 was not detected in *trim32* ^*-/-*^ MEF or *trim32* ^*-/-*^ cells reconstituted with the C39S mutant. The requirement for MG132 treatment to visualize PB1 ubiquitination suggests that PB1 undergoes degradation in the presence of TRIM32. Proteins modified with K48-linked polyubiquitin are classic targets for proteasomal degradation. To test this possibility, HEK293 cells were cotransfected with PB1 plus wild type ubiquitin or a ubiquitin construct in which all lysine residues except K48 were mutated to arginine (K48) and a ubiquitin construct in which only the K48 residue was mutated to arginine (K48R). After MG132 treatment wild type and K48-only ubiquitin were conjugated onto PB1, while ubiquitin molecules lacking the K48 residue (K48R) were not coupled onto PB1 (Fig 7C). Co-transfection with TRIM32 increased the levels of PB1 ubiquitination (Fig 7C). To examine the role of TRIM32 in PB1 protein turnover, GFP-TRIM32 and GFP-TRIM32(C39S) mutant constructs were transfected into A549 cells stably expressing FLAG-PB1. Ectopic expression of TRIM32 decreased PB1 expression, while TRIM32(C39S) showed little or no effect on PB1 expression (Fig 7D). To further examine the role of TRIM32 in PB1 degradation, wild type MEF, *trim32* ^-/-^ MEF and reconstituted *trim32* ^-/-^ MEF were treated with a protein synthesis inhibitor (cycloheximide). After 2 or 6 hr, cell lysates were examined by quantitative Western blotting. The data indicate the presence of TRIM32 has a destabilizing effect on PB1 expression (Fig 7E). Finally, we examined whether TRIM32-dependent PB1 ubiquitination had an effect on PB1 polymerase activity. TRIM32 transfection inhibits IAV polymerase activity (Fig 7F). As predicted, silencing TRIM32 with siRNA enhances polymerase activity, while addition of a RNAi resistant TRIM32 rescue construct reduced polymerase activity (Fig 7F). The combined data indicate that TRIM32 mediates K48-linked ubiquitination of PB1 resulting in augmented PB1 degradation and reduced viral polymerase activity.

## Discussion

Humans evolved a broad spectrum of defense strategies to limit IAV infection. Among them, the best characterized antiviral defense systems are the broadly acting TLR and RLR pattern-recognition receptors which detect microbial nucleic acids and other cross-species biomarkers. These innate antiviral defenses indirectly inhibit infection by triggering signaling cascades that lead to the production of interferons and other antiviral effector molecules. In contrast, as demonstrated in this study, TRIM32 operates in a more restricted fashion to directly sense an IAV “danger” signal and to restrict infection in a species specific manner. Thus, TRIM32 provides a form of defense often categorized as intrinsic immunity.

Examination of IAV polymerase interacting proteins by AP-MS identified TRIM32. Associations of TRIM32 with the PB1 subunit of the polymerase complex were noted using 6 distinct PB1 proteins derived from IAV strains of H1N1, H3N2, H5N1 and H7N9 origin, suggesting that PB1 has not yet adapted to avoid TRIM32 targeting. Preliminary data suggest TRIM32 conjugates polyubiquitin at multiple PB1 sites, thereby limiting the opportunity for PB1 mutants to avoid targeting by TRIM32.

The functional activity of TRIM32 was demonstrated by a combination of overexpression, mutagenesis and loss-of-function (RNAi and genetic deletion) analyses. Comparison between wild type TRIM32 and the C39S ligase defective mutant demonstrated the requirement for TRIM32 E3 ligase activity in antiviral activity. The efficacy of TRIM32 in restricting IAV infection was established with four IAV strains and multiple cell types, including a human lung epithelial cell line and primary human tracheal epithelial cells. The latter represent a model for host cells which are naturally targeted during IAV infection.

TRIM32 is expressed in all tissues and displays broad substrate specificity. TRIM32 is known to bind at least 10 different cellular proteins of highly diverse function and localization, most of these interaction partners are also known substrates of TRIM32-mediated ubiquitination [22,23,25–29]. TRIM32 is a remarkably versatile E3 ligase; it participates in monoubiquitination [20,25] and formation of K48-linked or K-63-linked polyubiquitin chains which are covalently conjugated to target proteins [29,30]. In addition, TRIM32 can form unanchored polyubiquitin chains [54]. Here we report that in response to influenza infection, TRIM32 targets PB1 for K48-linked ubiquitination. The K48 polyubiquitin tagged PB1 molecules are shuttled to the proteasome system which orchestrates turnover of target proteins.

Depending on context, some cell types predominantly express TRIM32 proteins in the nuclear compartment, while in other cell types it primarily resides in a cytoplasmic niche [23,24,27,34,55,56]. However, TRIM32 nuclear import and export sequences remain undefined. Leptomycin B treatment causes TRIM32 nuclear accumulation suggesting that TRIM32 shuttles between the cytosolic and nuclear compartments. TRIM32 trafficking was previously studied in neural stem cells, where TRIM32 autoubiquitination helps maintain cytoplasmic localization [24]. In another study, ubiquitin conjugating E2 enzymes controlled TRIM32 localization in nuclear or cytoplasmic compartments [57]. The current study suggests an additional mechanism capable of rapidly regulating TRIM32 localization. We propose that the increasing nuclear concentration of PB1 during IAV replication combined with the high affinity of TRIM32 for this substrate affords a mechanism to trap and accumulate TRIM32 in the nucleus. It would be interesting to learn if this trapping hypothesis also applies to other settings where TRIM32 accumulates in the nucleus.

Many distinct types of proteins can regulate antiviral responses and they can act at different stages of viral replication [31,52]. Among the most studied innate or intrinsic antiviral factors are members of the TRIM family. In fact TRIM32 was among the first TRIMs shown to specifically interact with a viral protein, tat [9]. Although TRIM32 binds to the activation domain of lentiviral tat proteins, the role of TRIM32 in viral restriction was not studied. In a separate report, TRIM32 was shown to target STING (stimulator of interferon genes) for ubiquitination [29]. TRIM32 also stimulates NF-κB activity [21]. Both of these pathways can lead to potentiation of type I IFN and other antiviral genes associated with innate immunity. However, genetic deficiency of TRIM32 does not significantly alter the levels of IFN production activated by flu infection. Many TRIM members require interferon to achieve expression and function [50]. In contrast, TRIM32 is constitutively expressed in respiratory epithelial cells where it is positioned to provide early and direct defense against IAV infection.

In summary, we demonstrate TRIM32 inhibits IAV polymerase activity, targets PB1 for proteasomal degradation and provides intrinsic cellular restriction against IAV infection. Exploitation of this natural defense pathway may offer potential strategies for controlling IAV infections. Currently approved treatments against influenza are losing effectiveness as new viral strains are often refractory to conventional treatments. Understanding the molecular mechanisms controlling intrinsic antiviral defenses may illuminate novel strategies for preventing and treating viral diseases.

## Materials and Methods

### Cell culture, stable cell lines and viruses

Primary human bronchial tracheal epithelial cells and supporting medium were purchased from Lifeline Cell Technology (Frederick, MD). HEK293 and A549 cells (American Type Culture Collection, Manassas, VA) were cultured in Dulbecco's modified Eagle's medium (DMEM) supplemented with 10% fetal bovine serum (FBS) in 10% CO_2_ at 37°C. *P65* ^*+/+*^ and *p65* ^*-/-*^ MEF were generously provided by Denis Guttridge (Ohio State University, Columbus, OH). Primary MEF derived from *trim32* ^+/+^ and *trim32* ^-/-^ mice [16] were transfected with SV40 large T antigen for immortalization. MEF were cultured in DMEM supplemented with 10% FBS. For generation of stable cell lines, human TRIM32 or mutant expression constructs were transfected into HEK293 cells, A549 cells and *trim32* ^-/-^ MEFs cells using Lipofectamine 2000 (Invitrogen, Carlsbad, CA) as detailed elsewhere [32]. Two days after transfection, cells were treated with 200 μg/ml hygromycin for 14 days. Single colonies were picked and expanded in 6-well plates. Protein expression levels in each colony were determined by immunoblotting. Influenza A viruses used in this study: A/Puerto Rico/8/34 (H1N1) (Charles River Labs, Wilmington, MA), A/PR8ΔNS1 (generously provided by Adolfo Garcia-Sastre, Mt. Sinai School of Medicine, NY) [53], A/WSN/33 (H1N1) (kindly provided by Peter Palese, Mt. Sinai School of Medicine, NY), A/New York/18/2009 (H1N1) (BEI Resources, Manassas, VA), and A/Aichi/68 (H3N2) (Charles River Labs). Influenza A PR8-GLuc virus containing a Gaussia luciferase (GLuc) gene inserted downstream of PB2 was a generous gift from Peter Palese [45]. Sendai virus was purchased from Charles River Labs. The Sendai-luciferase reporter virus was kindly provided by Charles Russell (St. Jude Hospital, Memphis, TN) [58].

### Purification of protein complexes and mass spectrometry

Mass spectrometry experiments were performed as previously described [38,39,59]. For protein purification HEK293 stable cell lines expressing FLAG-PB1 were collected from five 15 cm dishes in 10 ml TAP buffer (50 mM Tris-HCl [pH 7.5], 10 mM MgCl_2_, 100 mM NaCl, 0.5% Nonidet P40, 10% glycerol, phosphatase inhibitors and protease inhibitors) [32]. Cell lysates were precleared with 50 μl protein A/G resin before addition of 20 μl anti-FLAG resin (Sigma) and 16 hr incubation at 4°C on a rotator. The resin was 3X washed and transferred to a spin column (Sigma) with 40 μl 3X FLAG peptide (Sigma) for 1 hr at 4°C on a rotator. Purified complexes were loaded onto a 4–15% NuPAGE gel (Invitrogen). Gels were stained using the SilverQuest staining kit (Invitrogen) and lanes were excised for mass spectrometry analysis by the Taplin Biological Mass Spectrometry Facility (Harvard Medical School, Boston, MA).

### Interaction scoring for FLAG AP-MS

Two independent FLAG-PB1 purifications were analyzed by AP-MS. The resulting data are presented in S1 Table and were compared with our database of 200 controls from stable 293 cell lines expressing the FLAG tag fused to non-viral proteins handled in identical fashion. The SAINT algorithm (http://sourceforge.net/projects/saint-apms) was used to evaluate the MS data [41]. The default SAINT options were low Mode = 1, min Fold = 0, norm = 0. SAINT scores computed for each biological replicate were averaged (AvgP) and reported as the final SAINT score. Fold change was calculated for each prey protein as the ratio of spectral counts from replicate bait purifications over the spectral counts across all negative controls. A background factor of 0.1 was added to the average spectral counts of negative controls to prevent division by zero. Proteins included in the final interactome list had an AvgP ≥0.89. Selection of the threshold for SAINT scores was based on receiver operating curve analysis performed using publicly available protein interaction data and the FLAG AP-MS data set as a list of true positive interactions. A SAINT score of AvgP ≥0.89 was considered a true positive BioID protein with an estimated FDR of ≤2%.

### IAV propagation and titration

All influenza viruses were propagated in MDCK cells and specific pathogen-free (SPF) embryonated chicken eggs. Monolayers of MDCK cells were washed with phosphate-buffered saline (PBS) and incubated with the respective virus at a multiplicity of infection (MOI) of 0.001 at 37°C. After 1 hour, the inoculum was aspirated, cells were washed twice and incubated at 37°C with DMEM without serum supplemented with tosylsulfonyl phenylalanyl chloromethyl ketone (TPCK)-treated trypsin (1 μg/ml; Worthington Biomedical Corporation, Lakewood, NJ). 48 hr postinfection (p.i.) virus was recovered from supernatants. For SPF eggs, 0.2 ml stock influenza virus at 1x10^3^ TCID_50_ was injected into 11-day-old SPF fertile chicken eggs. The eggs were incubated in a stationary incubator at 35°C. After 72 hr incubation, eggs were cooled at -20°C for 30 min, then clear allantoic fluid was collected. For viral titration, plaque assays were performed as described [60]. Briefly, 1.2 × 10^6^ MDCK cells/ml were plated in 6-well plates. MDCK cells were washed twice with DMEM without serum, then serial dilutions of virus were adsorbed onto cells for 1 hr. Cells were then covered with DMEM 2×Avicel RC591 NF mix (FMC Biopolymer, Philadelphia, PA) supplemented with TPCK-treated trypsin (1 μg/ml). Crystal violet staining was performed 72 hr p.i., and visible plaques were counted.

### Antibodies, chemicals and plasmids

Monoclonal anti-FLAG (M2) and anti-HA antibodies were obtained from Sigma (St. Louis, MO). The polyclonal rabbit anti-TRIM32 was prepared as described elsewhere [23]. Mouse anti-PB1 and NP antibodies were obtained from BEI Resources. Anti-GFP antibody was purchased from Santa Cruz Biotechnology. The anti-lamin A, anti-α-tubulin and anti-ubiquitin antibodies were purchased from Cell Signaling Technology (Danvers, MA). Anti-β-actin was purchased from Abcam (Cambridge, MA). The anti-GST and anti-HIS antibodies were obtained from Bethyl Laboratories, Inc (Montgomery, TX). The 3X FLAG peptide, HA peptide, MG132 and cychlohexmide were purchased from Sigma. Poly(I:C) was purchased from Invivogen (San Diego, CA). Anti-Sendai virus was obtained from Charles River Labs. Anti-V5 antibody was bought from Thermo Scientific.

cDNA encoding full-length human TRIM32 or TRIM32 mutants were subcloned in frame into mammalian expression vector pCMV-3TAG-8 with a C-terminal 3XFLAG, V5 or HA and the pEGFP-N1 vector containing a C-terminal GFP. cDNA encoding full-length PB1 from PR8, WSN, Aichi, NY, H5N1 and H7N9 IAV (S2 Table) were subcloned into mammalian expression vector pCMV-3TAG-8 with a C-terminal 3XFLAG or HA tag. The cDNA encoding PR8 derived PA, PB2 or NP (S2 Table) were subcloned into mammalian expression vector pCMV-3TAG-8 with a C-terminal 3XFLAG or HA. The sources of all constructs are provided in S2 Table.

### Nuclear and cytoplasmic protein extraction

2x10^6^ A549 cells were prepared for nuclear and cytoplasmic protein extraction according to the manufacturer’s protocol (Pierce, Rockford, IL).

### Immunoprecipitation and immunoblotting

Immunoprecipitation and immunoblotting were performed as previously described [32]. For immunoprecipitation with anti-FLAG or anti-HA antibodies, the cell lysates were incubated with EZview red anti-FLAG M2 or anti-HA affinity resin (Sigma) for 4 or 16 hr at 4°C. After washing with lysis buffer, proteins were eluted by incubation with 1 mg/ml 3X FLAG or HA peptide for 1 hr at 4°C. For immunoprecipitation with anti-TRIM32 or anti-PB1, cell lysates were incubated with antibody and Protein A/G plus agarose (Thermo Fisher Scientific, Cambridge, MA) at 4°C for 16 hr. After washing with the lysis buffer, SDS-PAGE loading buffer was added and heated (95°C for 5 min). For immunoblotting, protein samples or 2% whole cell lysate were run on SDS-PAGE and transferred to PVDF membranes (Bio-Rad, Hercules, CA). The membranes were blocked in 5% non-fat milk in 1×Tris-buffered saline and then incubated with diluted primary antibodies at 4°C for 16 hr. Anti-rabbit or anti-mouse IgG antibodies conjugated to horseradish peroxidase (Pierce) were used as secondary antibodies. An enhanced chemiluminescence system (Pierce) was used for detection. Quantitation of immunoblots was performed using GelQuantNet software.

### Immunostaining

Cells were fixed with 4% formaldehyde in PBS for 10 min, permeabilized with methanol or 0.5% Triton X-100 for 15 min, blocked with 2% bovine serum albumin in PBS for 30 min, and then incubated with primary antibodies at 4°C for 16 hr. After three PBS washes, the cells were incubated with Alexa 488-labeled and/or Alexa 595-labeled secondary antibodies (Invitrogen) for 1 hr at room temperature. Cells were counterstained with DAPI (4',6-diamidino-2-phenylindole; Sigma). Automated imaging software (Fiji ImageJ) with colocalization 2 application package was used to quantitate colocalization.

### RNAi and rescue construct

FlexiTube siRNA oligos against TRIM32 were purchased from Qiagen (Valencia, CA). TRIM32 RNAi target sequences were as follows: #1 GACCGTGGTAACTATCGTATA, #2 CACACGATGGTGTTAGCTGAA and #3 CAGCACTCCAGGAATGTTCAA.

For siRNA gene knockdown experiments, cells were cultured in 24-well plates and transfected with 30 pmol siRNA and 3 μl lipofectamine 2000 according to the manufacturer’s instructions (Life Technologies, Grand Island, NY). After 48 hr, siRNA transfected cells were analyzed. A siRNA resistant TRIM32 was constructed in pCMV-3tag8-HA vector in which the No. 1 siRNA target sequence was mutated. Mutagenesis primers for the siRNA resistant rescue construct were as follows: 5’-tgaagtactagtcgctgatagaggaaagtacaggatccaagtctttacccgc-3’ and 5’-gcgggtaaagacttggatcctgtactttcctctatcagcgactagtacttca-3’

### Preparation of recombinant bacterial proteins

TRIM32-HIS was expressed from pET28b vector (Clontech Laboratories, Mountain View, CA) and PR8-PB1-GST was expressed from pGEX-5X-3 vector (GE Healthcare Life Sciences, Pittsburgh, PA) in Escherichia coli BL21(DE3) pLysS (Life Technologies) induced with 0.2 mM isopropyl-1-thio-β-D-galactopyranoside and 200 μM ZnSO_4_ for 16 hr at 18°C as detailed elsewhere [38]. GST-tagged proteins were purified with glutathione Sepharose 4B beads according to the manufacturer’s protocol (GE Healthcare Life Sciences). HIS-tag proteins were purified with Ni-NTA agarose resins according to the manufacturer’s protocol (Qiagen).

### Real-time quantitative PCR analysis for gene expression

Total RNA was extracted with RNeasy mini kit (Invitrogen) and reverse-transcribed (2 μg) with QuantiTect Reverse Transcription Kit (Qiagen). Human TRIM32, human IFNβ, human GAPDH, mouse IFNβ and mouse β-glucuronidase mRNA levels were quantitated by RT-PCR with SYBR dyes on a LightCycler 480 (Roche Life Sciences) as described elsewhere [38,61]. Primers for hTRIM32 were: forward CCGGGAAGTGCTAGAATGCC and reverse CAGCGGACACCATTGATGCT.

### *In vitro* ubiquitination assays

*In vitro* ubiquitination assays were performed according to the manufacturer’s manual (Boston Biochem, Cambridge, MA). Ubiquitin (5 μg), E1 (200 ng), UBCH5a (300 ng) (Boston Biochem), TRIM32-HIS (0.8 μg) and PR8-PB1-GST (2 μg) were incubated with 2 mM ATP (Sigma) at 37°C 2 hr in ubiquitin assay buffer (20 mM Tris-HCl pH7.5, 5 mM MgCl_2_, 2 mM DTT). 1X stop solution (Boston Biochem) was added to end the reaction, after GST pull down the sample was washed with 1 M Urea for 60 min to exclude potential binding of unanchored polyubiquitin, then the sample was placed in SDS-loading buffer and boiled at 95°C for 5 min. Samples were subsequently analyzed by SDS-PAGE followed by Western blotting.

### Reporter assay

Cells were aliquoted in 24 well plates for 24 hr prior to IAV infection. Human primary and cell lines were infected with 0.01 MOI PR8-GLuc and MEF were infected with 0.1MOI. After 1 hr the viral inoculum was aspirated, cells were washed twice and incubated at 37°C with 0.2% BSA-DMEM without serum. Cells were lysed 12–16 hr p.i. and the luciferase assay was performed using BioLux Gaussia Luciferase Assay Kit (NEB, Ipswich, MA).

### Reconstitution of influenza virus polymerase activity

HEK293 cells were transfected in triplicate with vectors expressing PR8 PB1, PB2, NP, PA and the indicated TRIM32 or siTRIM32 oligo in addition to the polymerase I (PolI)-driven plasmid transcribing an influenza A virus-like RNA coding for the reporter protein firefly luciferase to monitor viral polymerase activity [62]. Cells were lysed 48 hr after transfection. Luciferase activity was measured with a luciferase assay system (Promega, Madison, WI). A plasmid constitutively expressing Renilla luciferase was transfected as a control.

### Statistical analysis

Unless indicated otherwise all experiments were repeated on at least three separate occasions. Data from representative experiments are illustrated. Methods for AP-MS data analysis are detailed elsewhere [41]. Other statistical analyses were done with the two-tailed Student’s *t* test. Data are presented as mean ± standard deviation. A *P* value of <0.05 was considered significant.

### Accession numbers

Identification of the genes and proteins used throughout this study include: TRIM32 (BC003154), TRIM65 (BC021259.2), STUB1 (BC0075456), IQSEC (BC010267), GALK1 (BC 001166), PDCD6 (BC050597) and DGT6/HAUS6 (NM 001270890.1). The GenBank references for viral genes include: PR8 PB1 (EF467819), WSN PB1 (J02178.1), NY PB1 (CY039907.1), Aichi PB1 (CY121123), H5N1 PB1 (AY651664.1), H7N9 PB1 (CY147058.1), H7N9 PB1 (GISAID # EPI439508) PR8 PB2 (CY148250.1), PR8 PA (CY148248.1) and PR8 NP (CY148246.1).

## Supporting Information

S1 Fig TRIM32 interacts with influenza A virus PB1. (A) HEK293 cells were transfected with FLAG conjugated TRIM32, STUB1 and IQSEC1 or GFP-coupled TRIM32, GALK1, PDCD6 and DGT6/HAUS6. After 24 hr, cells were infected with 0.01 MOI PR8-Gluc for 16 hr. The relative luciferase signal is shown. An asterisk indicates P<0.01. Bottom panels display transfection efficiency by Western blot. (B) PB1 interacts with endogenous TRIM32 in HEK293 cells. 12 hr postinfection (p.i.) with PR8 IAV, whole cell lysates (WCL) were subjected to immunoprecipitation (IP) and immunoblotting with indicated antibodies. (C) Full length TRIM32 fused with HA epitope was co-transfected with PR8 derived FLAG-PB1 and PA into HEK293 cells. After 48 hr, WCL were immunoprecipitated with anti-FLAG antibody and blotted with indicated reagents. (D) A549 cells were infected with PR8 strain IAV for 4 hr or stimulated with 100 U/ml IFNβ for 4 hr. Cells were collected for mRNA extraction. Real-time PCR was performed to detect TRIM32 mRNA levels relative to GAPDH controls. (E) A549 cells were infected with PR8 strain IAV for 16 hr or treated with 100 U/ml IFNβ for 16 hr. Western blot of cell lysates were probed with indicated antibodies. Quantitative Western blotting was used to calculate relative TRIM32 protein levels.
(TIF)

S2 Fig TRIM32 nuclear translocation. (A) A549 cells were infected with 0.01 MOI PR8 IAV for the indicated times and stained with anti-PB1 (red), anti-TRIM32 (green) and DAPI nuclear stain (blue). Right panel shows quantitated TRIM32-PB1 colocalization data. (B) A549 control or A549 cells stably expressing FLAG-PB1 were stained with anti-PB1 (red), anti-TRIM32 (green) and DAPI nuclear stain (blue). (C) A549 cells were treated with the indicated dose of leptomycin B for 2 hr. After fixation, cells were stained with anti-TRIM32 (red) and DAPI nuclear stain (blue). (D) PB1 and PB2 were cotransfected along with TRIM32 into HEK293 cells. The indicated antibodies were used for immunoprecipitation and blotting.
(TIF)

S3 Fig TRIM32 restricts influenza virus infection. (A) A549 cells transfected with vector or TRIM32. After 48 hr, cell viability was assessed by exclusion of trypan blue. (B) A549 stable cell lines reconstituted with control vector or TRIM32-FLAG were infected with indicated MOI of IAV PR8 for 16 hr. WCL were blotted with indicated antibodies. (C) HEK293 cells were transfected with FLAG-TRIM32. After 24 hr, cells were infected with indicated MOI of WSN strain IAV for 18 hr. Cells were collected for Western blot with indicated antibodies. (D) HEK293 cells were transfected with GFP or TRIM32-GFP. After 24 hr, cells were infected with different MOI of IAV PR8 for 16 hr. Cell lysates were Western blotted with indicated antibodies. (E) A549 stable cell lines transfected with control vector or TRIM32-FLAG were infected with WSN strain IAV for 8 hr, then fixed and stained with anti-NP (red) plus DAPI. The right panel shows the relative ratio of NP stained cells. Asterisk indicates P<0.01. (F) A549 cells were transiently transfected with GFP or TRIM32-GFP. After 24 hr cells were infected with indicated MOI of WSN IAV for 24 hr. Supernatant was titered on MDCK cells and plaques were enumerated. Asterisk indicates P<0.05. (G) A549 stable cell lines transfected with control vector or TRIM32-FLAG were infected with the indicated IAV strains (0.001 MOI) for 16 hr. Then, 10 μl supernatant was transferred to another plate of A549 cells. After 16 hr, target cells were fixed and stained with anti-NP. Pooled data from two experiments. NP stained cells from five random fields were counted. The relative fraction of infected cells ± SD is presented. An asterisk indicates P<0.01.
(TIF)

S4 Fig TRIM32 deficiency promotes IAV infection. (A) A549 cells were transfected with scrambled control siRNA or 3 individual TRIM32 siRNA duplexes. After 24 hr cells were infected with 0.01 MOI PR8-Gluc for 16 hr. The relative luciferase activity was examined. An asterisk indicates P<0.01. Right panel displays knockdown efficiency by Western blot. (B) A549 cells were transfected with control or TRIM32-siRNA. After 24 hr cells were infected with indicated MOI of WSN IAV for 24 hr. Supernatant was titered on MDCK cells and pfu were enumerated. Asterisk indicates P<0.05. (C) A549 cells were transfected with control or TRIM32 siRNA. After 24 hr cells were infected with 0.01 MOI PR8 for 8 hr, cells were then fixed and stained with anti-TRIM32 (green), anti-NP (red) and DAPI (blue) for microscopic analysis. The lower panel displays the relative ratio of TRIM32 and NP stained cells. An asterisk indicates P<0.01. (D) *Trim32* ^+/+^ and *trim32* ^-/-^ MEF were infected with 0.1 MOI of the indicated IAV strain. After 16 hr, medium from MEF was plated onto A549 cells to compare the levels of virus production. A549 cells were stained with anti-NP, and relative numbers of NP stained cells were calculated. Data are from one of two similar experiments. Five different fields were counted. Asterisk indicates P<0.01.
(TIF)

S5 Fig Influenza A virus induced IFNβ production is independent of TRIM32. (A) *Trim32* ^+/+^ and *trim32* ^-/-^ MEF were treated with PR8 IAV ΔNS1. Cells were collected for mRNA extraction. Real-time PCR was performed to detect IFNβ and control β-glucuronidase mRNA. Relative IFNβ mRNA expression is depicted. (B) *Trim32* ^+/+^ and *trim32* ^-/-^ MEF were treated with 2 μg/ml poly(I:C) for 2–4 hr. Cells were collected for mRNA extraction. Realtime PCR was performed to detect IFNβ and control β-glucuronidase mRNA. Relative IFNβ mRNA expression is depicted. (C) *P65* ^+/+^ and *p65* ^-/-^ MEF were infected with PR8-Gluc for 12 hr. The relative Gaussia luciferase signal is shown.
(TIF)

S1 Table Affinity purification coupled with mass spectrometry identifies PB1 interacting proteins. FLAG-PB1 (PR8) was affinity purified from stably transfected HEK293 cells. Purified PB1 protein complexes from two independent experiments were analyzed by mass spectrometry. Spectral counts from each experiment are listed. SAINT score (≥0.89) was used to identify high confidence interacting proteins (HCIP) which are highlighted in yellow. 18 of 26 HCIP confirm associations noted from previous reports.
(XLSX)

S2 Table List of plasmids. Source of plasmids used in this study.
(XLSX)
