# Supplementary figures and images for: TRIM32 Senses and Restricts Influenza A Virus by Ubiquitination of PB1 Polymerase
Source: PLoS Pathog. 2015 Jun 9;11(6):e1004960. doi: 10.1371/journal.ppat.1004960 (PMC4461266; doi:10.1371/journal.ppat.1004960)

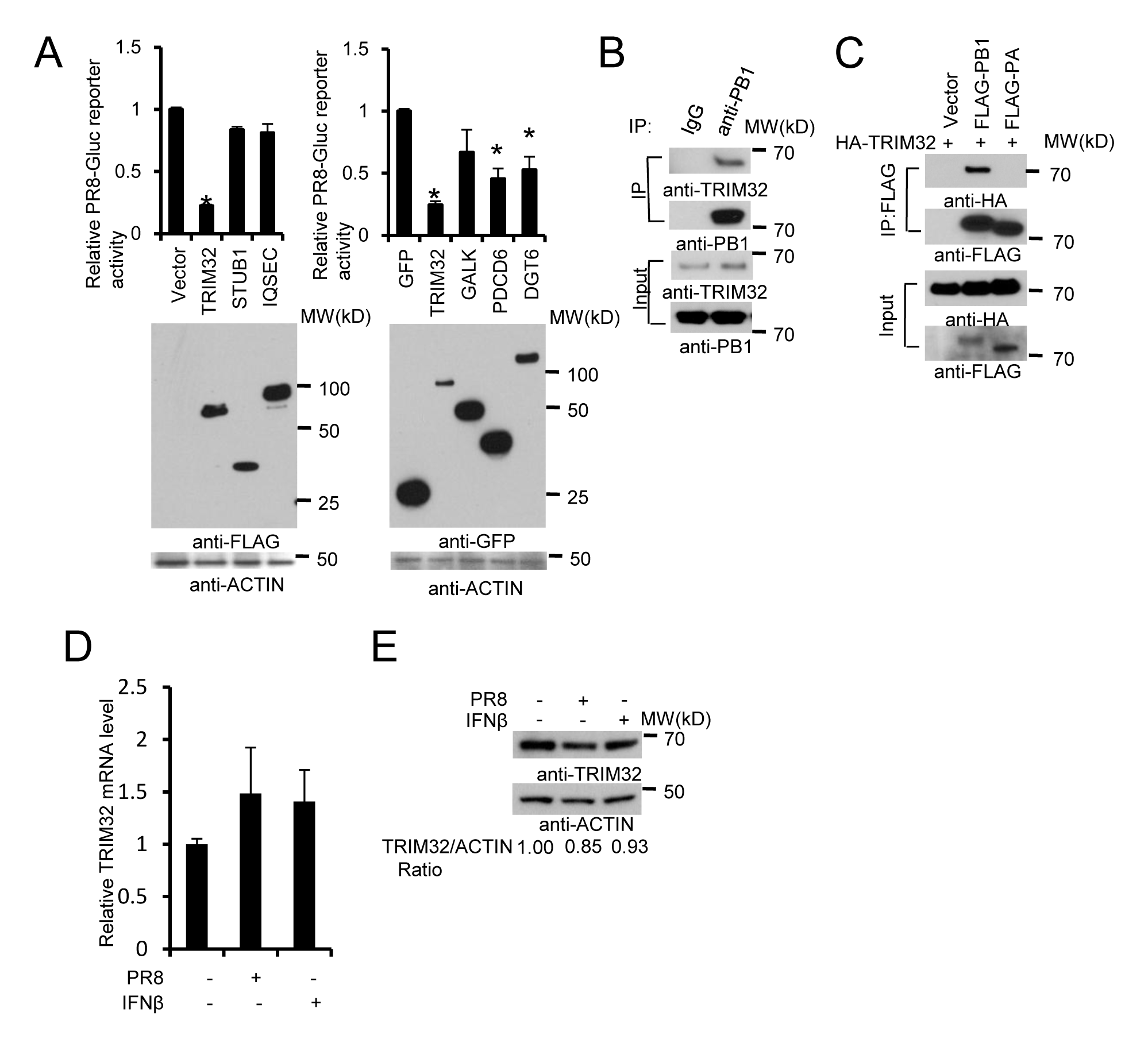

Supplement: S1 Fig — (A) HEK293 cells were transfected with FLAG conjugated TRIM32, STUB1 and IQSEC1 or GFP-coupled TRIM32, GALK1, PDCD6 and DGT6/HAUS6. After 24 hr, cells were infected with 0.01 MOI PR8-Gluc for 16 hr. The relative luciferase signal is shown. An asterisk indicates P<0.01. Bottom panels display transfection efficiency by Western blot. (B) PB1 interacts with endogenous TRIM32 in HEK293 cells. 12 hr postinfection (p.i.) with PR8 IAV, whole cell lysates (WCL) were subjected to immunoprecipitation (IP) and immunoblotting with indicated antibodies. (C) Full length TRIM32 fused with HA epitope was co-transfected with PR8 derived FLAG-PB1 and PA into HEK293 cells. After 48 hr, WCL were immunoprecipitated with anti-FLAG antibody and blotted with indicated reagents. (D) A549 cells were infected with PR8 strain IAV for 4 hr or stimulated with 100 U/ml IFNβ for 4 hr. Cells were collected for mRNA extraction. Real-time PCR was performed to detect TRIM32 mRNA levels relative to GAPDH controls. (E) A549 cells were infected with PR8 strain IAV for 16 hr or treated with 100 U/ml IFNβ for 16 hr. Western blot of cell lysates were probed with indicated antibodies. Quantitative Western blotting was used to calculate relative TRIM32 protein levels. (TIF) [file ppat.1004960.s001.tif]

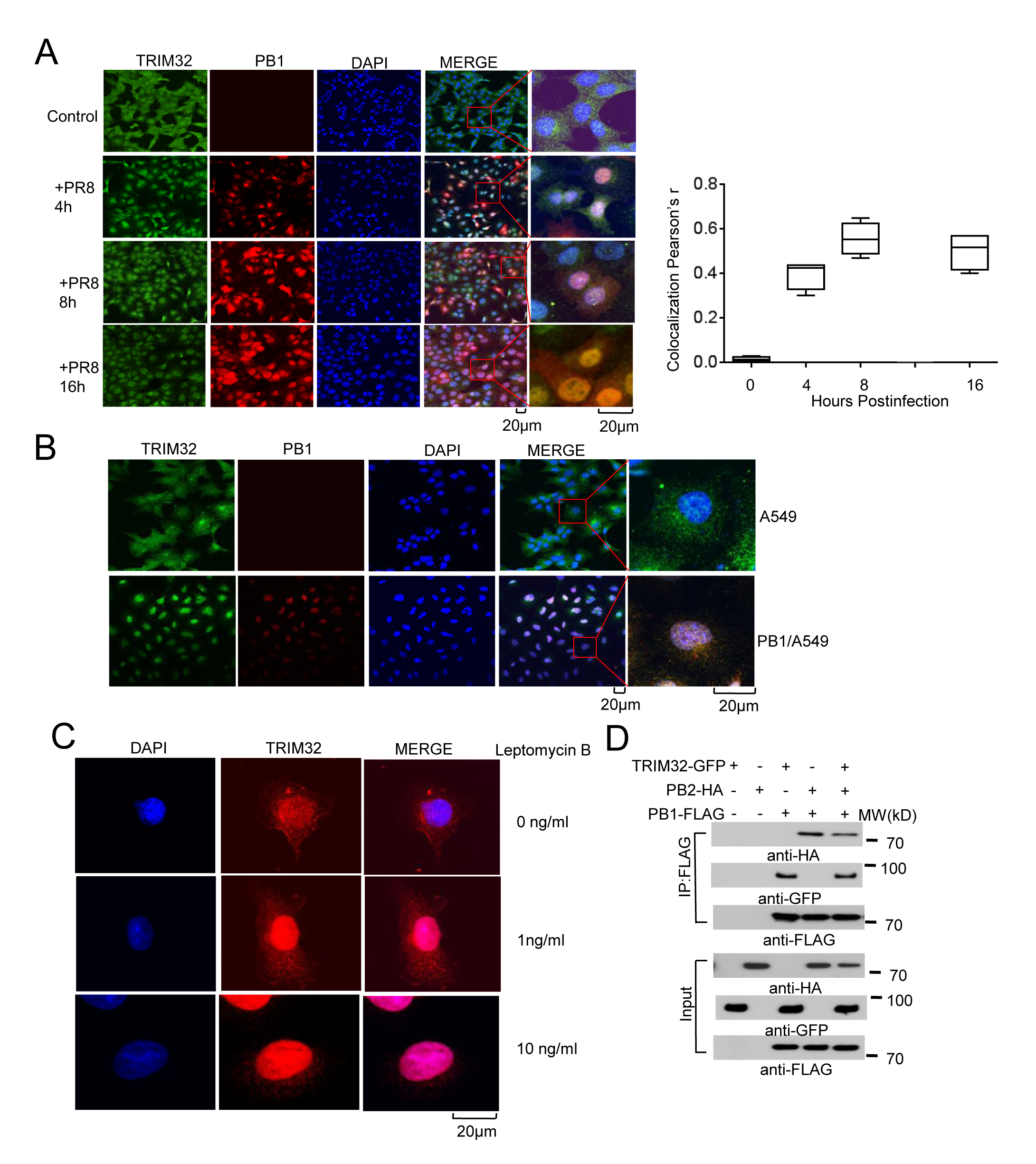

Supplement: S2 Fig — (A) A549 cells were infected with 0.01 MOI PR8 IAV for the indicated times and stained with anti-PB1 (red), anti-TRIM32 (green) and DAPI nuclear stain (blue). Right panel shows quantitated TRIM32-PB1 colocalization data. (B) A549 control or A549 cells stably expressing FLAG-PB1 were stained with anti-PB1 (red), anti-TRIM32 (green) and DAPI nuclear stain (blue). (C) A549 cells were treated with the indicated dose of leptomycin B for 2 hr. After fixation, cells were stained with anti-TRIM32 (red) and DAPI nuclear stain (blue). (D) PB1 and PB2 were cotransfected along with TRIM32 into HEK293 cells. The indicated antibodies were used for immunoprecipitation and blotting. (TIF) [file ppat.1004960.s002.tif]

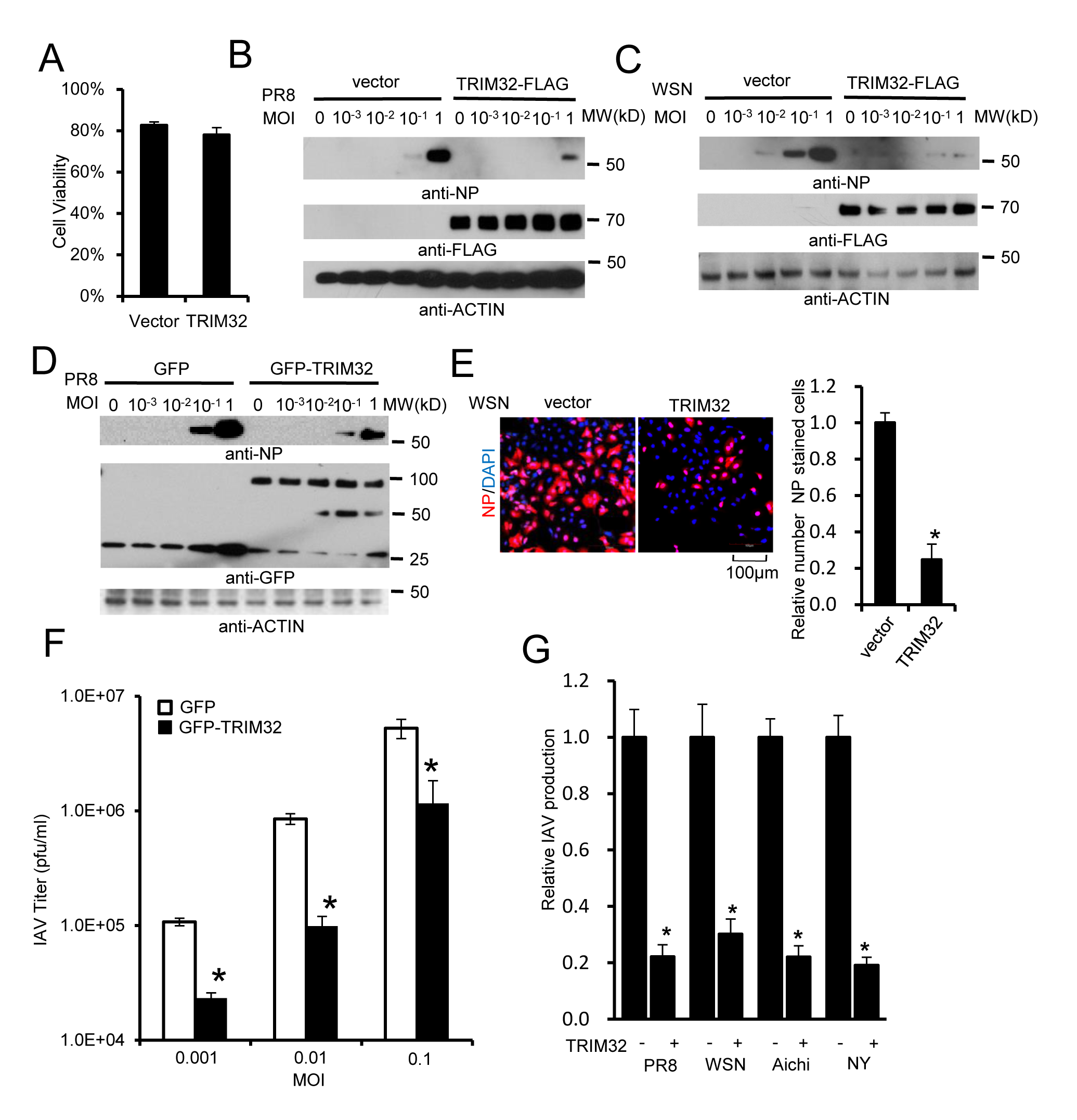

Supplement: S3 Fig — (A) A549 cells transfected with vector or TRIM32. After 48 hr, cell viability was assessed by exclusion of trypan blue. (B) A549 stable cell lines reconstituted with control vector or TRIM32-FLAG were infected with indicated MOI of IAV PR8 for 16 hr. WCL were blotted with indicated antibodies. (C) HEK293 cells were transfected with FLAG-TRIM32. After 24 hr, cells were infected with indicated MOI of WSN strain IAV for 18 hr. Cells were collected for Western blot with indicated antibodies. (D) HEK293 cells were transfected with GFP or TRIM32-GFP. After 24 hr, cells were infected with different MOI of IAV PR8 for 16 hr. Cell lysates were Western blotted with indicated antibodies. (E) A549 stable cell lines transfected with control vector or TRIM32-FLAG were infected with WSN strain IAV for 8 hr, then fixed and stained with anti-NP (red) plus DAPI. The right panel shows the relative ratio of NP stained cells. Asterisk indicates P<0.01. (F) A549 cells were transiently transfected with GFP or TRIM32-GFP. After 24 hr cells were infected with indicated MOI of WSN IAV for 24 hr. Supernatant was titered on MDCK cells and plaques were enumerated. Asterisk indicates P<0.05. (G) A549 stable cell lines transfected with control vector or TRIM32-FLAG were infected with the indicated IAV strains (0.001 MOI) for 16 hr. Then, 10 μl supernatant was transferred to another plate of A549 cells. After 16 hr, target cells were fixed and stained with anti-NP. Pooled data from two experiments. NP stained cells from five random fields were counted. The relative fraction of infected cells ± SD is presented. An asterisk indicates P<0.01. (TIF) [file ppat.1004960.s003.tif]

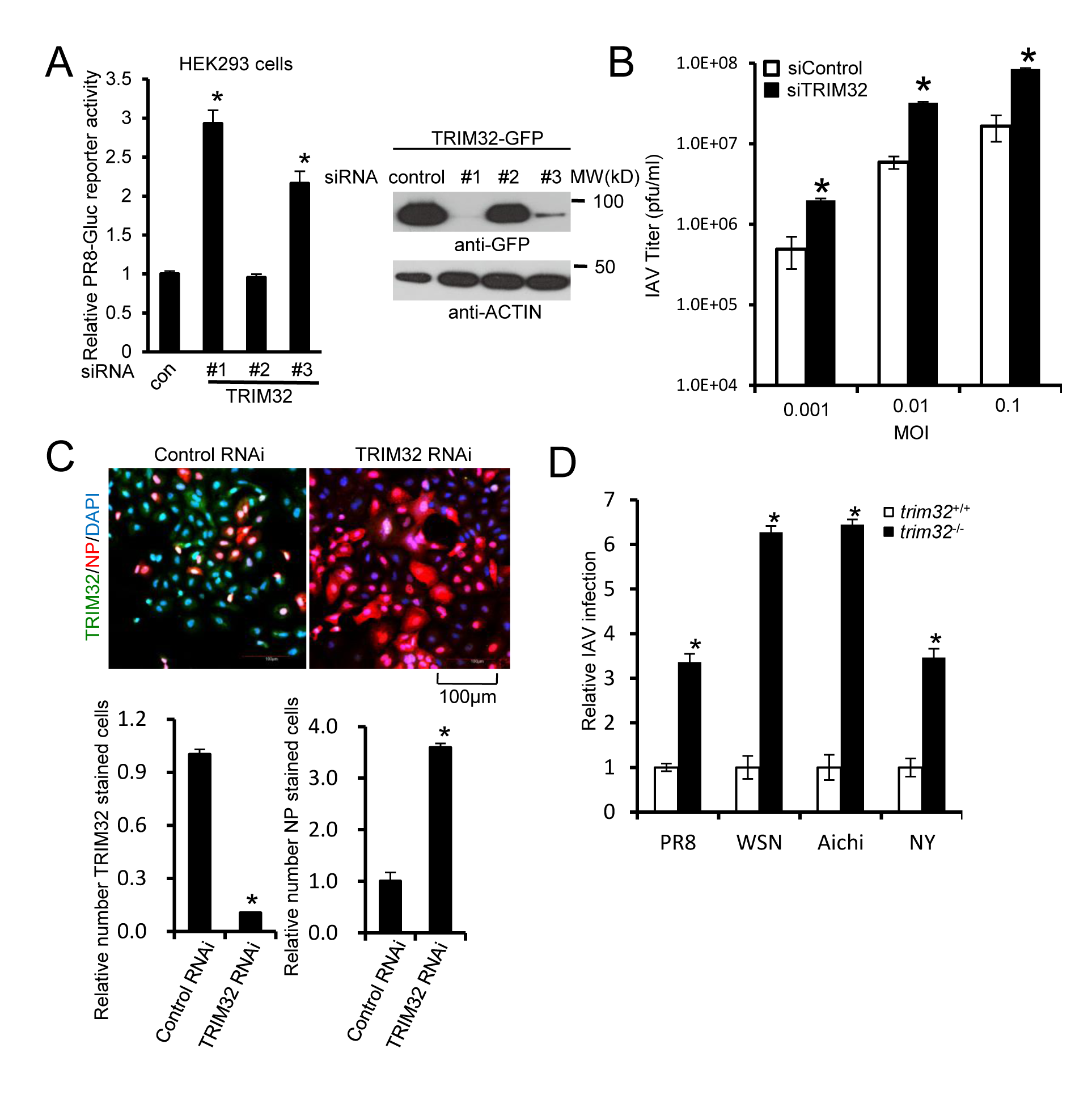

Supplement: S4 Fig — (A) A549 cells were transfected with scrambled control siRNA or 3 individual TRIM32 siRNA duplexes. After 24 hr cells were infected with 0.01 MOI PR8-Gluc for 16 hr. The relative luciferase activity was examined. An asterisk indicates P<0.01. Right panel displays knockdown efficiency by Western blot. (B) A549 cells were transfected with control or TRIM32-siRNA. After 24 hr cells were infected with indicated MOI of WSN IAV for 24 hr. Supernatant was titered on MDCK cells and pfu were enumerated. Asterisk indicates P<0.05. (C) A549 cells were transfected with control or TRIM32 siRNA. After 24 hr cells were infected with 0.01 MOI PR8 for 8 hr, cells were then fixed and stained with anti-TRIM32 (green), anti-NP (red) and DAPI (blue) for microscopic analysis. The lower panel displays the relative ratio of TRIM32 and NP stained cells. An asterisk indicates P<0.01. (D) Trim32 +/+ and trim32 -/- MEF were infected with 0.1 MOI of the indicated IAV strain. After 16 hr, medium from MEF was plated onto A549 cells to compare the levels of virus production. A549 cells were stained with anti-NP, and relative numbers of NP stained cells were calculated. Data are from one of two similar experiments. Five different fields were counted. Asterisk indicates P<0.01. (TIF) [file ppat.1004960.s004.tif]

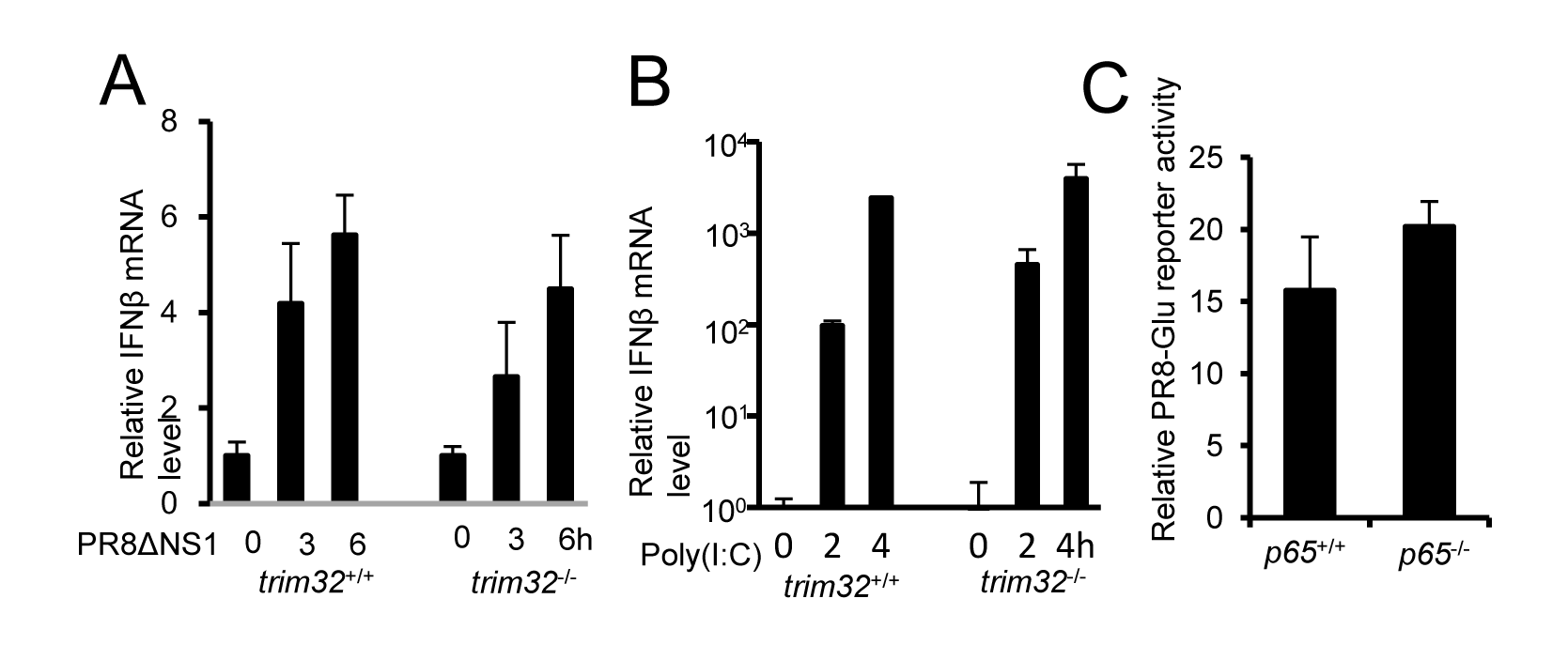

Supplement: S5 Fig — (A) Trim32 +/+ and trim32 -/- MEF were treated with PR8 IAV ΔNS1. Cells were collected for mRNA extraction. Real-time PCR was performed to detect IFNβ and control β-glucuronidase mRNA. Relative IFNβ mRNA expression is depicted. (B) Trim32 +/+ and trim32 -/- MEF were treated with 2 μg/ml poly(I:C) for 2–4 hr. Cells were collected for mRNA extraction. Realtime PCR was performed to detect IFNβ and control β-glucuronidase mRNA. Relative IFNβ mRNA expression is depicted. (C) P65 +/+ and p65 -/- MEF were infected with PR8-Gluc for 12 hr. The relative Gaussia luciferase signal is shown. (TIF) [file ppat.1004960.s005.tif]
